# The association between systemic inflammation, lung function and respiratory symptoms in the BOLD study in Northern Europe

**DOI:** 10.1038/s41598-025-16776-x

**Published:** 2025-08-22

**Authors:** Lucia Cestelli, Andre F. S. Amaral, Bryndis Benediktsdottir, Thorarinn Gislason, Rain Jõgi, Andrei Malinovschi, Rune Nielsen, Christer Janson

**Affiliations:** 1 Department of Clinical Science, University of Bergen, PO Box 7804, Bergen, 5020 Norway; 2 Department of Global Public Health and Primary Care, University of Bergen, Bergen, Norway; 3 National Heart and Lung Institute, Imperial College London, London, UK; 4 Department of Sleep, Landspitali University Hospital, Reykjavik, Iceland; 5 Faculty of Medicine, University of Iceland, Reykjavik, Iceland; 6 The Lung Clinic, Tartu University Hospital, Tartu, Estonia; 7 Department of Medical Sciences: Clinical Physiology, Uppsala University, Uppsala, Sweden; 8 Department of Thoracic Medicine, Haukeland University Hospital, Bergen, Norway; 9 Department of Medical Sciences: Respiratory, Allergy and Sleep Research, Uppsala University, Uppsala, Sweden

**Keywords:** Inflammation, White blood cells, Spirometry, Lung function, Smoking, Respiratory symptoms, Asthma, Chronic obstructive pulmonary disease, Epidemiology, Biomarkers, Inflammation, Respiratory signs and symptoms

## Abstract

The role of systemic inflammation in the pathogenesis of respiratory diseases is increasingly recognized, but the relationship between individual inflammatory markers, lung function and respiratory symptoms is not well established. We studied 1,238 adults participating in the first follow-up of the Burden of Obstructive Lung Disease (BOLD) cohort study in Sweden, Norway, Iceland, and Estonia in 2019–2021. Systemic inflammation was assessed using total white blood cell (WBC) count and WBC sub-populations (neutrophils, lymphocytes, monocytes, eosinophils, basophils and neutrophil-to-lymphocyte ratio (NLR)). In regression models adjusted for gender, age, smoking status, body mass index and study site, all WBC sub-populations, except for lymphocytes, were associated with chronic airflow obstruction (CAO) and wheeze. In never smokers, increased neutrophils were associated with reduced lung volumes (FEV_1_ β coef. with 95%CI -1.95 (-3.33, -0.57) *p* = 0.006; FVC − 1.94 (-3.18, -0.69) *p* = 0.002), but not CAO. Only increased basophils were associated with CAO in never smokers (OR with 95%CI 2.70 (1.28, 5.72) *p* = 0.009). In former smokers, increased neutrophils, monocytes and eosinophils were significantly associated with reduced FEV_1_, reduced FVC, CAO and wheeze. In current smokers, inflammatory markers were associated with cough (neutrophils OR with 95%CI 1.49 (1.10, 2.01) *p* = 0.010; monocytes 1.24 (0.99, 1.55) *p* = 0.058; basophils 2.56 (1.12, 5.86) *p* = 0.026). Systemic inflammation may be related to both obstructive and restrictive respiratory impairment in the general population, with associations that vary by smoking status.

**Supplementary Information:**

The online version contains supplementary material available at 10.1038/s41598-025-16776-x.

## Introduction

Systemic inflammation is involved in the pathogenesis of several respiratory diseases^1–4^. White blood cell (WBC) total count and sub-populations are easily obtainable biomarkers that can reflect systemic inflammation.

In cross-sectional and longitudinal studies, blood eosinophils have been associated with reduced lung function^5–7^. They are considered to have a pathogenetic role in type 2-high asthma and, to a lesser extent, chronic obstructive pulmonary disease (COPD)^8^. Blood neutrophils have been associated with lower forced expiratory volume in the 1 st second (FEV_1_), forced vital capacity (FVC), and FEV_1_/FVC in the general population^5^, and severity and phenotype of asthma and COPD in clinical studies^9,10^.

Few studies have explored the relationship between other WBC sub-populations and lung function in general population samples, and the findings were not conclusive^11–13^. Two studies reported inverse associations of blood monocytes and basophils with FEV_1_^11,13^, while another study found an association with monocytes but not basophils^12^. Some have also reported a relationship between blood lymphocytes and reduced FEV_1_^12,13^, but not others^11^. Limited information is also available regarding the association between systemic inflammation and respiratory symptoms^11,14,15^.

Smoking status is an important factor to consider in these associations. Higher blood counts of eosinophils, neutrophils and monocytes have been described in current smokers^16–18^ and cigarette smoking is the major determinant of COPD and related symptoms such as cough, phlegm and dyspnoea. Increased WBCs, however, can also be found in asthma and it is increasingly recognized that COPD and reduced lung volumes can be present in never smokers^19,20^.

The main aim of this study was to investigate the association of circulating WBCs with lung function and respiratory symptoms in adults from four Northern European countries participating in the first follow-up of the Burden of Obstructive Lung Disease (BOLD) cohort study. We hypothesized the presence of distinct profiles of associations between inflammatory markers, lung function indices (FEV_1_, FVC, FEV_1_/FVC, chronic airflow obstruction) and respiratory symptoms (cough, phlegm, wheezing, dyspnoea), and that these associations vary by smoking status (current, former, never smokers).

## Methods

### Study population

The BOLD cohort study was designed to estimate the prevalence of chronic obstructive lung disease and identify its risk factors across several world regions^21,22^. At baseline, representative samples of non-institutionalized adults aged 40 years or older were identified from the general population around sites with at least 150,000 inhabitants. Participants were selected from a random sample of the population according to a predefined site-specific sampling strategy. The current analysis is based on data collected in four North European sites [Uppsala (Sweden), Bergen (Norway), Reykjavik (Iceland), Tartu (Estonia)] between 2019 and 2021 during the first follow-up of the participants seen at baseline. Participants provided information on demographics, smoking habits and respiratory symptoms through a standardized questionnaire and performed spirometry to test their lung function. In the four North European sites, participants also provided blood samples. Participants were included in this study if they had completed the core study questionnaire and had availability of the blood sample. Participants were excluded if they had died, emigrated, were institutionalized, could not be contacted, actively refused to participate or had contraindications for lung function testing. The number of participants at the baseline and follow-up stages is reported in Table S1. All sites received approval from their local ethics committee and all participants provided informed consent. The follow-up study was also approved by Imperial College London Research Ethics Committee (ref. 17IC4272). The research was conducted in accordance with the Declaration of Helsinki.

### Respiratory symptoms

Respiratory symptoms consisted of cough and phlegm in the absence of cold (defined as affirmative answer to the questions “Do you usually cough when you don’t have a cold?” and “Do you usually bring up phlegm from your chest, or do you usually have phlegm in your chest that is difficult to bring up when you don’t have a cold?”, chronic cough and chronic phlegm (“Do you cough on most days for as much as three months each year?” and “Do you bring up this phlegm on most days for as much as three months each year?”), wheeze (“Have you had wheezing or whistling in your chest at any time in the last 12 months?”), and dyspnoea (defined as ≥ 2 on the modified Medical Research Council (mMRC) dyspnoea scale). These definitions were based on the standardized BOLD questionnaire used in previous research^21,23^.

### Lung function

Spirometry was performed with a ndd EasyOne spirometer (ndd Medizintechnik, Zurich, Switzerland) before and after bronchodilation with 200 micrograms of salbutamol through a spacer. All spirometry curves were checked centrally at the BOLD Operations Centre, and to be considered useable, tests had to include at least three acceptable curves (no hesitation, complete blow, no artefact affecting lung function readings), with the two best blows being within 200mL of each other. Predicted values of FEV_1_, FVC, and FEV_1_/FVC were calculated using NHANES reference equations^24^. Airflow obstruction was defined as FEV_1_/FVC < LLN (lower limit of normal, defined as 5th percentile) according to ERS/ATS 2005 guidelines^25^. We used post-bronchodilatory values to define chronic airflow obstruction (CAO) in order to exclude subjects with reversible airflow obstruction.

### White blood cell (WBC) counts

Blood samples were collected to measure inflammatory markers. Total WBC and WBC sub-populations (neutrophils, lymphocytes, monocytes, eosinophils, and basophils) were measured with automated hematological analyzers. The neutrophil-to-lymphocyte ratio (NLR) was the ratio between neutrophils and lymphocytes^26^.

### Statistical analysis

Data in the descriptive analyses was presented as mean ± SD, median (IQR) and %, as appropriate. We compared variables across study sites with ANOVA for continuous normally distributed variables, Kruskal-Wallis test for continuous non-normally distributed variables and chi-square test for categorical variables. Multivariable linear regression was used to analyze the association between inflammatory markers and post-bronchodilatory FEV_1_, FVC and FEV_1_/FVC % of predicted as continuous variables. Beta coefficients with 95% confidence intervals (CI) indicate the average change in lung function per one-unit increase of inflammatory markers. Multivariable logistic regression was used to analyze the association of inflammatory markers with CAO and respiratory symptoms (categorical variables). Odds ratios (ORs) with 95% CI indicate the odds of the outcome per one-unit increase of inflammatory markers.

All analyses were adjusted for gender, age (continuous), smoking status (categorized as never, former, current), body mass index (BMI, categorized as < 18.5, 18.5–24.9, 25.0–29.9, ≥ 30.0 kg/m^2^) and study site. We further investigated the role of smoking status in the association between inflammatory markers and outcomes using stratified analyses and interaction tests. In supplementary material, we reported the results of the unadjusted analyses. In sensitivity analyses, we tested if the associations between inflammatory markers, lung function and respiratory symptoms were comparable across study sites by performing random-effects meta-analysis. Missing data on covariates were minimal (smoking status *n* = 9, BMI *n* = 4) and they were not imputed. Analyses were conducted on complete data. Stata version 18 (StataCorp LLC) was used to analyze the data.

## Results

### Characteristics of the study population

We obtained data from 1,238 participants. The mean age was 68 years and there were slightly more men (53%) than women. Of the participants, *n* = 533 (43%) were never smokers, *n* = 548 (45%) were former smokers and *n* = 148 (12%) were current smokers (Table 1). About 10% had CAO, one in five reported wheezing within the last 12 months, and chronic cough was reported by one in ten. Mean total WBC were 6.1±2.1 × 10^9^/L and median blood eosinophils were 0.18 (0.13) x 10^9^/L.

**Table 1:** Characteristics of the participants in the four North European sites of the Burden of Obstructive Lung Disease follow-up study.

Demographics/Anthropometrics						
	All *n* = 1238	Reykjavik *n* = 371	Bergen *n* = 296	Uppsala *n* = 266	Tartu *n* = 305	p-value
Age (years)	68.1 ± 8.9	66.8 ± 8.3	69.6 ± 8.8	68.0 ± 8.4	68.3 ± 9.7	< 0.001
Women	46.9	45.6	46.6	42.9	52.5	0.121
Smoking status						< 0.001
Never	43.4	40.2	34.8	43.6	55.4	
Former	44.6	49.1	49.7	49.4	30.2	
Current	12.0	10.8	15.5	7.0	14.4	
BMI (kg/m^2^)						< 0.001
< 18.5	0.3	0.3	0.0	0.0	1.0	
18.5–24.9	27.0	19.1	38.5	33.2	20.0	
25–29.9	42.2	43.4	42.2	44.7	38.7	
≥ 30	30.5	37.2	19.3	22.1	40.3	

Blood inflammatory markers						
	*n* = 1238	*n* = 371	*n* = 296	*n* = 266	*n* = 305	p-value
Total WBC (10^9^/L)	6.1 ± 2.1	5.9 ± 2.3	6.1 ± 2.4	6.5 ± 2.1	6.1 ± 1.6	0.014
Neutrophils (10^9^/L)	3.4 ± 1.2	3.1 ± 1.0	3.4 ± 1.2	3.7 ± 1.3	3.5 ± 1.2	< 0.001
Lymphocytes (10^9^/L)	1.9 ± 1.5	2.0 ± 1.9	1.9 ± 1.7	1.9 ± 1.3	1.8 ± 0.6	0.772
NLR	2.0 ± 1.0	1.8 ± 0.8	2.0 ± 1.0	2.2 ± 1.1	2.1 ± 1.0	< 0.001
Monocytes (10^9^/L)	0.53 ± 0.16	0.51 ± 0.15	0.52 ± 0.17	0.55 ± 0.18	0.52 ± 0.15	0.009
Eosinophils (10^9^/L)	0.18 (0.13)	0.2 (0.2)	0.2 (0.2)	0.13 (0.12)	0.15 (0.12)	< 0.001
Basophils (10^9^/L)	0.03 (0.08)	0.0 (0.10)	0.0 (0.10)	0.04 (0.03)	0.04 (0.04)	< 0.001

Post-bronchodilatory lung function						
	*n* = 1056	*n* = 337	*n* = 276	*n* = 239	*n* = 204	p-value
FEV_1_ (%pred)	95.3 ± 16.5	92.1 ± 15.8	96.0 ± 15.9	95.4 ± 16.6	99.5 ± 17.1	< 0.001
FVC (%pred)	96.0 ± 14.2	94.2 ± 13.8	96.7 ± 13.8	94.0 ± 14.4	100.5 ± 14.1	< 0.001
FEV_1_/FVC (%pred)	99.0 ± 9.9	97.5 ± 10.6	99.0 ± 10.1	101.1 ± 8.0	98.7 ± 10.2	< 0.001
CAO	10.1	12.5	10.5	4.6	12.2	0.011

Respiratory symptoms						
	*n* = 1229	*n* = 371	*n* = 296	*n* = 257	*n* = 305	p-value
Cough w/o cold	26.8	34.8	22.0	26.8	21.6	< 0.001
Chronic cough	9.0	13.0	6.8	7.9	7.2	0.014
Phlegm w/o cold	18.2	17.8	20.9	17.5	16.7	0.558
Chronic phlegm	9.3	11.0	10.5	8.2	6.9	0.232
Wheeze	20.7	24.3	19.6	18.7	19.3	0.255
mMRC ≥ 2	6.0	6.9	2.3	7.9	6.7	0.051

Data are presented as mean ± SD, median (IQR) or %, as appropriate. BMI = body mass index, WBC = white blood cell, NLR = neutrophil-lymphocyte ratio, FEV_1_ = forced expiratory volume in the 1 st second, FVC = forced vital capacity, CAO = chronic airflow obstruction, defined as FEV_1_/FVC values below 5th percentile, mMRC = modified Medical Research Council dyspnoea scale.

Characteristics stratified by smoking status are reported in Tables S2–S4. The total WBC, neutrophils, lymphocytes, monocytes, eosinophils and basophils counts were higher in current smokers, followed by former smokers and never smokers. Current smokers had lower FEV_1_, FVC and FEV_1_/FVC than former and never smokers and a higher prevalence of respiratory symptoms and CAO (25.8% vs. 10.3% vs. 5.8%).

### Inflammatory markers and lung function

**Fig. 1:**
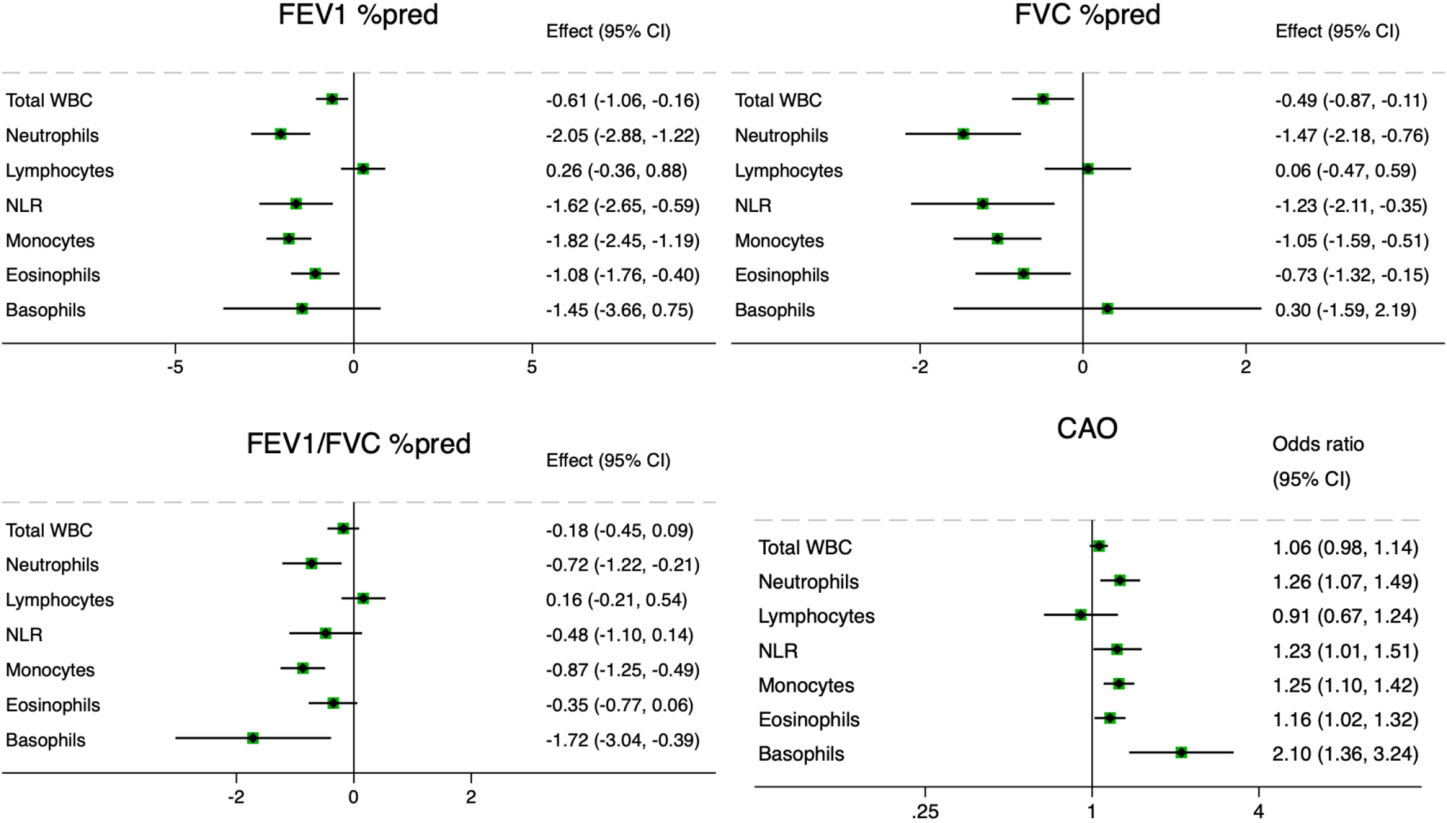
Associations between inflammatory markers and post-bronchodilatory lung function in the total population (β coefficients and odds ratios (OR) per unit with 95% confidence intervals (CI) adjusted for gender, age, body mass index, smoking status and study site). Abbreviations: FEV_1_ = forced expiratory volume in the 1 st second, FVC = forced vital capacity, CAO = chronic airflow obstruction (defined as post-bronchodilatory FEV_1_/FVC values below 5th percentile), WBC = white blood cells, NLR = neutrophil-lymphocyte ratio. Units of measurement: total WBC, neutrophils, lymphocytes 10^9^/L; monocytes, eosinophils, basophils 10^10^/L.

In the total population (Fig. 1), increased neutrophils (OR with 95%CI 1.26 (1.07, 1.49) *p* = 0.005), monocytes (1.25 (1.10, 1.42) *p* = 0.001) and eosinophils (1.16 (1.02, 1.32) *p* = 0.022) were positively associated with CAO and negatively associated with FEV_1_ and FVC. Increased basophils were positively associated with CAO (OR with 95%CI 2.10 (1.36, 3.24) *p* = 0.001) and negatively associated with FEV_1_ (β coef. with 95%CI −1.45 (−3.66, 0.75) *p* = 0.196), but not with FVC (0.30 (−1.59, 2.19) *p* = 0.756).

When stratified by smoking status (Table 2), in never-smokers, increased neutrophils and the NLR were associated with reduced FEV_1_ (β coef. with 95%CI −1.95 (−3.33, −0.57) *p* = 0.006, and − 2.14 (−3.79, −0.50) *p* = 0.011, respectively) and reduced FVC (β coef. with 95%CI −1.94 (−3.18, −0.69) *p* = 0.002, and − 1.62 (−3.10, −0.13) *p* = 0.033, respectively), but not CAO. Only increased basophils were associated with CAO (OR with 95%CI 2.70 (1.28, 5.72) *p* = 0.009) in never-smokers. In former smokers, multiple inflammatory markers (total WBC, neutrophils, monocytes, eosinophils) were significantly associated with reduced FEV_1_, reduced FVC, and CAO. A similar tendency was observed in current smokers, although these associations did not reach statistical significance likely due to the small size of the subgroup.

**Table 2:** Associations between inflammatory markers and post-bronchodilatory lung function stratified by smoking status (β coefficients and odds ratios (OR) per unit with 95% confidence intervals (CI) adjusted for gender, age, body mass index and study site).

	**FEV**_**1**_ **%pred**			**FVC %pred**				**FEV**_**1**_ **/FVC %pred**		**CAO**	
	Coef. (95% CI)		p-value	Coef. (95% CI)		p-value		Coef. (95% CI)	p-value	OR (95% CI)	p-value
*Never smokers*											
Total WBC (10^9^/L)	−0.01 (−0.54, 0.52)	0.979		−0.16 (−0.64, 0.31)		0.499		0.14 (−0.14, 0.42)	0.316	0.98 (0.83, 1.15)	0.810
Neutrophils (10^9^/L)	**−1.95 (−3.33**,** −0.57)**	**0.006**		**−1.94 (−3.18**,** −0.69)**		**0.002**		−0.03 (−0.76, 0.70)	0.938	1.14 (0.78, 1.69)	0.493
Lymphocytes (10^9^/L)	0.50 (−0.13, 1.13)	0.120		0.24 (−0.33, 0.81)		0.409		0.24 (−0.09, 0.58)	0.149	0.63 (0.29, 1.39)	0.253
NLR	**−2.14 (−3.79**,** −0.50)**	**0.011**		**−1.62 (−3.10**,** −0.13)**		**0.033**		−0.54 (−1.41, 0.33)	0.223	1.33 (0.86, 2.05)	0.195
Monocytes (10^10^/L)	**−1.32 (−2.31**,** −0.33)**	**0.009**		−0.84 (−1.73, 0.06)		0.067		**−0.53 (−1.05**,** −0.01)**	**0.045**	1.23 (0.94, 1.61)	0.123
Eosinophils (10^10^/L)	−0.52 (−1.45, 0.41)	0.274		−0.39 (−1.23, 0.45)		0.360		−0.15 (−0.63, 0.34)	0.555	1.07 (0.86, 1.32)	0.544
Basophils (10^10^/L)	−2.98 (−6.31, 0.34)	0.078		−0.84 (−3.84, 2.17)		0.585		**−1.96 (−3.70**,** −0.23)**	**0.027**	**2.70 (1.28**,** 5.72)**	**0.009**
*Former smokers*											
Total WBC (10^9^/L)	**−2.15 (−3.10**,** −1.21)**	**< 0.001**		**−1.34 (−2.08**,** −0.60)**		**< 0.001**		**−1.00 (−1.59**,** −0.41)**	**0.001**	**1.25 (1.05**,** 1.48)**	**0.013**
Neutrophils (10^9^/L)	**−2.33 (−3.53**,** −1.13)**	**< 0.001**		**−1.41 (−2.35**,** −0.47)**		**0.003**		**−1.19 (−1.93**,** −0.44)**	**0.002**	**1.32 (1.07**,** 1.63)**	**0.010**
Lymphocytes (10^9^/L)	−2.16 (−4.71, 0.39)	0.096		−1.58 (−3.57, 0.40)		0.118		−0.63 (−2.21, 0.94)	0.431	0.94 (0.56, 1.59)	0.830
NLR	−1.36 (−2.84, 0.11)	0.070		−0.94 (−2.09, 0.21)		0.109		−0.62 (−1.53, 0.29)	0.181	1.26 (0.98, 1.63)	0.075
Monocytes (10^10^/L)	**−2.38 (−3.32**,** −1.44)**	**< 0.001**		**−1.38 (−2.12**,** −0.64)**		**< 0.001**		**−1.15 (−1.73**,** −0.56)**	**< 0.001**	**1.28 (1.06**,** 1.54)**	**0.009**
Eosinophils (10^10^/L)	**−1.86 (−2.99**,** −0.74)**	**0.001**		**−1.00 (−1.89**,** −0.12)**		**0.026**		**−0.85 (−1.55**,** −0.15)**	**0.018**	**1.34 (1.10**,** 1.64)**	**0.004**
Basophils (10^10^/L)	0.79 (−2.63, 4.21)	0.650		2.00 (−0.66, 4.66)		0.140		−1.07 (−3.18, 1.04)	0.320	1.70 (0.88, 3.26)	0.113
*Current smokers*											
Total WBC (10^9^/L)	−1.62 (−3.32, 0.07)	0.061		−0.99 (−2.67, 0.70)		0.248		−0.78 (−2.11, 0.56)	0.250	1.23 (0.93, 1.62)	0.145
Neutrophils (10^9^/L)	−1.76 (−4.02, 0.50)	0.125		−0.97 (−3.21, 1.27)		0.393		−0.93 (−2.70, 0.84)	0.303	1.28 (0.88, 1.87)	0.195
Lymphocytes (10^9^/L)	−3.14 (−7.74, 1.47)	0.180		−2.29 (−6.84, 2.26)		0.321		−1.37 (−4.98, 2.23)	0.452	1.33 (0.65, 2.69)	0.433
NLR	−0.85 (−4.05, 2.35)	0.599		−0.97 (−4.12, 2.17)		0.541		0.18 (−2.32, 2.67)	0.888	1.05 (0.64, 1.71)	0.860
Monocytes (10^10^/L)	−1.42 (−3.11, 0.27)	0.098		−0.73 (−2.41, 0.94)		0.388		−0.77 (−2.09, 0.55)	0.251	1.27 (0.96, 1.67)	0.089
Eosinophils (10^10^/L)	−1.14 (−3.53, 1.24)	0.345		−1.45 (−3.79, 0.89)		0.223		0.39 (−1.47, 2.25)	0.680	1.06 (0.72, 1.54)	0.780
Basophils (10^10^/L)	−4.46 (−10.54, 1.62)	0.149		−1.63 (−7.66, 4.40)		0.594		−3.53 (−8.27, 1.20)	0.142	2.38 (0.88, 6.44)	0.088

FEV_1_ = forced expiratory volume in the 1 st second, FVC = forced vital capacity, CAO = chronic airflow obstruction, WBC = white blood cells, NLR = neutrophil-lymphocyte ratio. CI of beta coefficients not including zero and CI of ORs not including 1 are in bold.

### Inflammatory markers and respiratory symptoms

**Fig. 2:**
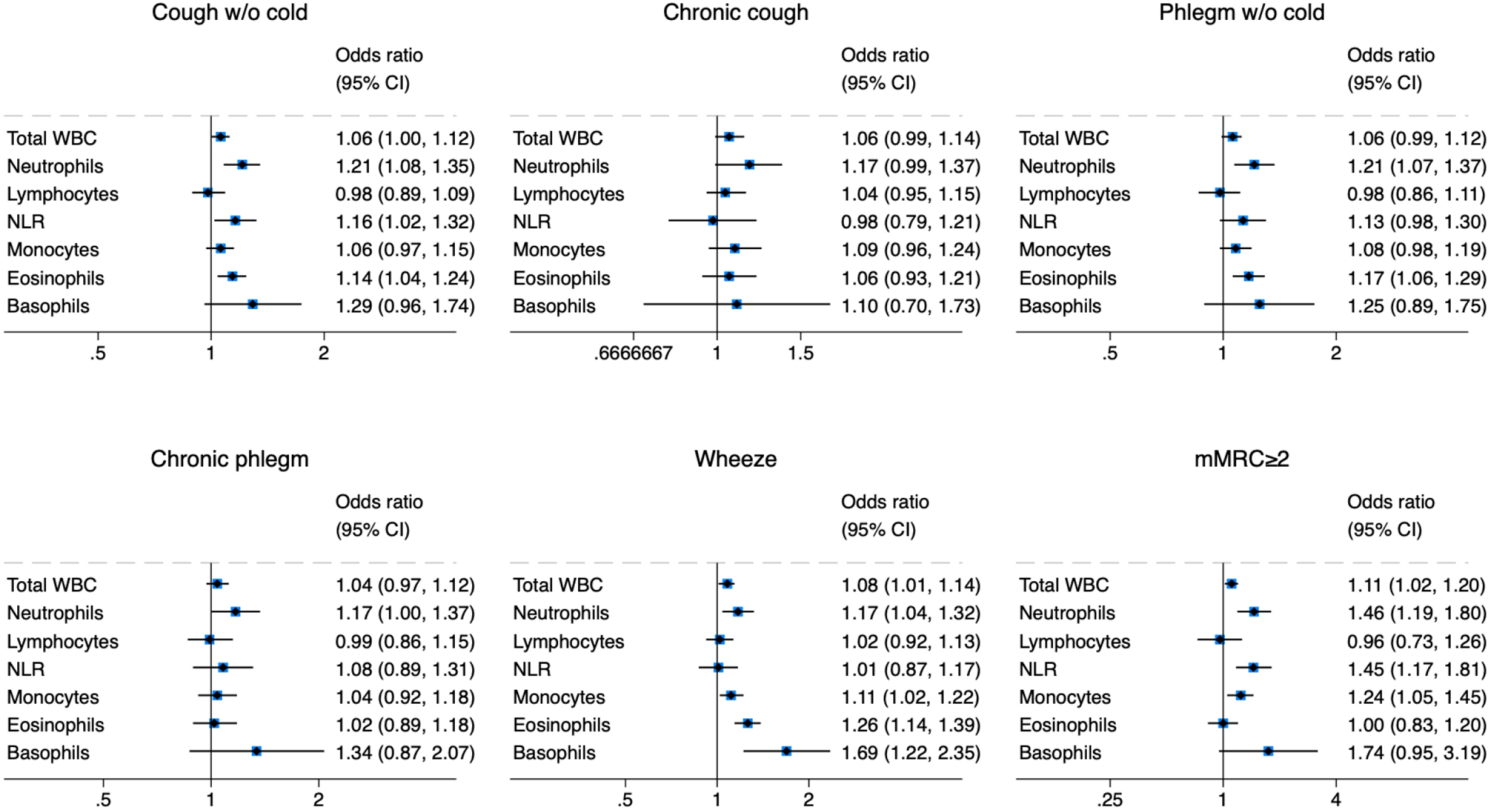
Associations between inflammatory markers and respiratory symptoms in the total population (odds ratios (OR) per unit with 95% confidence intervals (CI) adjusted for gender, age, body mass index, smoking status and study site). Abbreviations: mMRC = modified Medical Research Council dyspnoea scale, WBC = white blood cells, NLR = neutrophil-lymphocyte ratio. Units of measurement: total WBC, neutrophils, lymphocytes 10^9^/L; monocytes, eosinophils, basophils 10^10^/L.

In the total population (Fig. 2), increased neutrophils were associated with presence of all respiratory symptoms (cough and phlegm in the absence of a cold, chronic cough, chronic phlegm, wheeze and dyspnoea). Increased eosinophils were associated with cough in the absence of a cold (OR with 95%CI 1.14 (1.04, 1.24) *p* = 0.005), phlegm in the absence of a cold (1.17 (1.06, 1.29) *p* = 0.002), and wheeze (1.26 (1.14, 1.39) *p* < 0.001). Increased basophils were associated with cough in the absence of a cold (1.29 (0.96, 1.74) *p* = 0.097), wheeze (1.69 (1.22, 2.35) *p* = 0.002), and dyspnoea (1.74 (0.95, 3.19) *p* = 0.073).

When stratified by smoking status (Table 3), associations of inflammatory markers with respiratory symptoms were more numerous in current and former smokers compared to never smokers. In never smokers, increased neutrophils and the NLR were associated with cough in the absence of a cold and dyspnoea, while increased eosinophils were associated with cough and phlegm in the absence of a cold. In former smokers, all inflammatory markers, except for lymphocytes, were associated with wheeze, and neutrophils were associated with all respiratory symptoms. In current smokers, inflammatory markers were predominantly associated with cough and phlegm, especially basophils (OR with 95%CI: chronic cough 3.98 (0.89, 17.86) *p* = 0.071, chronic phlegm 6.12 (1.36, 27.56) *p* = 0.018).

**Table 3:** Associations between inflammatory markers and respiratory symptoms stratified by smoking status (odds ratios (OR) per unit with 95% confidence intervals (CI) adjusted for gender, age, body mass index and study site).

	Cough w/o cold		Chronic cough		Phlegm w/o cold		Chronic phlegm		Wheeze		mMRC ≥ 2	
	OR (95%CI)	p-value	OR (95%CI)	p-value	OR (95%CI)	p-value	OR (95%CI)	p-value	OR (95%CI)	p-value	OR (95%CI)	p-value
*Never smokers*												
Total WBC (10^9^/L)	1.06 (1.00, 1.12)	0.069	1.04 (0.95, 1.15)	0.360	0.99 (0.88, 1.12)	0.896	1.00 (0.86, 1.16)	0.993	1.01 (0.90, 1.12)	0.894	1.06 (0.91, 1.24)	0.449
Neutrophils (10^9^/L)	**1.21 (1.08**,** 1.35)**	**0.001**	1.07 (0.80, 1.45)	0.639	0.97 (0.75, 1.25)	0.787	0.99 (0.71, 1.39)	0.960	0.93 (0.73, 1.18)	0.564	**1.57 (1.07**,** 2.29)**	**0.021**
Lymphocytes (10^9^/L)	0.98 (0.89, 1.09)	0.744	1.04 (0.94, 1.16)	0.440	0.97 (0.78, 1.19)	0.759	1.00 (0.83, 1.20)	0.994	1.01 (0.89, 1.16)	0.840	0.84 (0.50, 1.38)	0.483
NLR	**1.16 (1.02**,** 1.32)**	**0.025**	0.82 (0.55, 1.22)	0.323	0.90 (0.66, 1.22)	0.501	0.80 (0.51, 1.25)	0.331	0.73 (0.53, 1.02)	0.063	**1.58 (1.07**,** 2.33)**	**0.022**
Monocytes (10^10^/L)	1.06 (0.97, 1.15)	0.180	1.16 (0.94, 1.42)	0.162	1.11 (0.93, 1.32)	0.261	1.15 (0.92, 1.44)	0.231	1.06 (0.90, 1.25)	0.514	1.13 (0.86, 1.49)	0.376
Eosinophils (10^10^/L)	**1.14 (1.04**,** 1.24)**	**0.005**	1.05 (0.86, 1.28)	0.642	**1.25 (1.08**,** 1.43)**	**0.002**	0.96 (0.74, 1.24)	0.744	**1.23 (1.07**,** 1.42)**	**0.003**	0.95 (0.72, 1.26)	0.720
Basophils (10^10^/L)	1.29 (0.96, 1.74)	0.097	0.74 (0.33, 1.67)	0.469	0.60 (0.29, 1.25)	0.172	**0.33 (0.11**,** 1.03)**	**0.055**	1.25 (0.68, 2.28)	0.475	1.30 (0.45, 3.73)	0.631
*Former smokers*												
Total WBC (10^9^/L)	1.09 (0.97, 1.22)	0.158	**1.16 (1.00**,** 1.35)**	**0.058**	**1.14 (1.01**,** 1.29)**	**0.032**	1.14 (0.98, 1.32)	0.081	**1.26 (1.11**,** 1.44)**	**< 0.001**	**1.25 (1.04**,** 1.50)**	**0.020**
Neutrophils (10^9^/L)	**1.20 (1.02**,** 1.41)**	**0.026**	**1.29 (1.05**,** 1.59)**	**0.015**	**1.33 (1.12**,** 1.57)**	**0.001**	**1.33 (1.09**,** 1.62)**	**0.005**	**1.36 (1.14**,** 1.62)**	**0.001**	**1.57 (1.19**,** 2.07)**	**0.002**
Lymphocytes (10^9^/L)	0.92 (0.70, 1.20)	0.526	1.01 (0.73, 1.42)	0.932	0.85 (0.60, 1.20)	0.353	0.86 (0.55, 1.34)	0.505	1.12 (0.90, 1.39)	0.327	0.97 (0.64, 1.45)	0.871
NLR	1.17 (0.98, 1.41)	0.088	1.15 (0.89, 1.49)	0.278	**1.29 (1.07**,** 1.56)**	**0.009**	**1.27 (1.01**,** 1.60)**	**0.043**	1.08 (0.89, 1.32)	0.430	**1.45 (1.09**,** 1.94)**	**0.012**
Monocytes (10^10^/L)	1.02 (0.90, 1.16)	0.726	1.02 (0.85, 1.23)	0.848	1.03 (0.90, 1.18)	0.619	0.98 (0.82, 1.16)	0.778	**1.15 (1.01**,** 1.31)**	**0.036**	**1.32 (1.04**,** 1.67)**	**0.024**
Eosinophils (10^10^/L)	1.10 (0.95, 1.27)	0.200	1.12 (0.91, 1.37)	0.275	1.09 (0.93, 1.27)	0.289	1.09 (0.90, 1.33)	0.371	**1.37 (1.17**,** 1.60)**	**< 0.001**	1.04 (0.77, 1.40)	0.811
Basophils (10^10^/L)	1.06 (0.68, 1.67)	0.788	1.10 (0.57,2.14)	0.769	1.24 (0.77, 2.00)	0.384	**1.78 (0.98**,** 3.25)**	**0.058**	**2.11 (1.31**,** 3.41)**	**0.002**	1.81 (0.74, 4.40)	0.193
*Current smokers*												
Total WBC (10^9^/L)	**1.40 (1.11**,** 1.78)**	**0.005**	0.99 (0.69, 1.42)	0.952	**1.32 (1.04**,** 1.68)**	**0.021**	1.00 (0.72, 1.40)	0.998	1.07 (0.86, 1.33)	0.558	1.08 (0.68, 1.72)	0.749
Neutrophils (10^9^/L)	**1.49 (1.10**,** 2.01)**	**0.010**	0.82 (0.50, 1.37)	0.456	1.28 (0.95, 1.73)	0.109	0.82 (0.52, 1.30)	0.399	1.07 (0.80, 1.42)	0.650	1.15 (0.61, 2.19)	0.667
Lymphocytes (10^9^/L)	1.76 (0.97, 3.21)	0.064	1.92 (0.72, 5.12)	0.195	**2.04 (1.08**,** 3.88)**	**0.029**	1.91 (0.80, 4.53)	0.143	1.00 (0.56, 1.79)	0.994	0.80 (0.24, 2.70)	0.719
NLR	1.28 (0.86, 1.91)	0.226	0.51 (0.18, 1.45)	0.207	1.14 (0.74, 1.75)	0.563	0.73 (0.35, 1.52)	0.405	1.18 (0.79, 1.78)	0.415	1.65 (0.85, 3.19)	0.138
Monocytes (10^10^/L)	**1.24 (0.99**,** 1.55)**	**0.058**	1.07 (0.73, 1.56)	0.724	1.16 (0.92, 1.46)	0.214	1.04 (0.74, 1.46)	0.805	1.14 (0.91, 1.41)	0.254	1.27 (0.77, 2.09)	0.345
Eosinophils (10^10^/L)	0.94 (0.70, 1.27)	0.682	0.81 (0.44, 1.47)	0.486	1.17 (0.86, 1.60)	0.308	0.91 (0.58, 1.44)	0.686	1.26 (0.93, 1.70)	0.141	1.10 (0.58, 2.06)	0.776
Basophils (10^10^/L)	**2.56 (1.12**,** 5.86)**	**0.026**	**3.98 (0.89**,** 17.86)**	**0.071**	**6.27 (2.10**,** 18.73)**	**0.001**	**6.12 (1.36**,** 27.56)**	**0.018**	1.89 (0.85, 4.22)	0.121	4.44 (0.77, 25.63)	0.095

mMRC = modified Medical Research Council dyspnea scale, WBC = white blood cells, NLR = neutrophil-lymphocyte ratio. CI of ORs not including 1 are in bold.

### Interactions

We examined the interaction of smoking status in the association between inflammatory markers and outcomes in Tables S5 and S6. A significant interaction was identified in the association between total WBC and all lung function indices, with former smokers having larger negative associations of total WBC with FEV_1_ (*p* < 0.001) and FVC (*p* = 0.012), and larger positive associations with CAO (*p* = 0.030) (Table S5). A similar interaction was also found in the association of lymphocytes (*p* = 0.044) and eosinophils (*p* = 0.038) with FEV_1_.

With regard to respiratory symptoms (Table S6), we found a significant interaction of smoking status in the association between total WBC and cough and phlegm without a cold (larger positive effect in current smokers, *p* = 0.005 and *p* = 0.046, respectively). Another significant interaction was found in the association of total WBC (*p* = 0.007), neutrophils (*p* = 0.007) and the NLR (*p* = 0.025) with wheeze, with larger positive effects in former smokers. Finally, there were significant interactions in the association between basophils and chronic cough and chronic phlegm (larger positive effect in current smokers, *p* = 0.043 and *p* = 0.002, respectively).

### Unadjusted analyses

The main analyses were adjusted for gender, age, body mass index and study site. Unadjusted analyses of the association between inflammatory markers, lung function and respiratory symptoms are reported in Table S7 and S8. The results were overall consistent with the adjusted analyses.

### Variation across study sites

The associations of inflammatory markers with lung function (Figures S1–S8) and respiratory symptoms (not shown) calculated with random-effect meta-analysis demonstrated non-significant low to moderate heterogeneity across study sites.

## Discussion

**Fig. 3:**
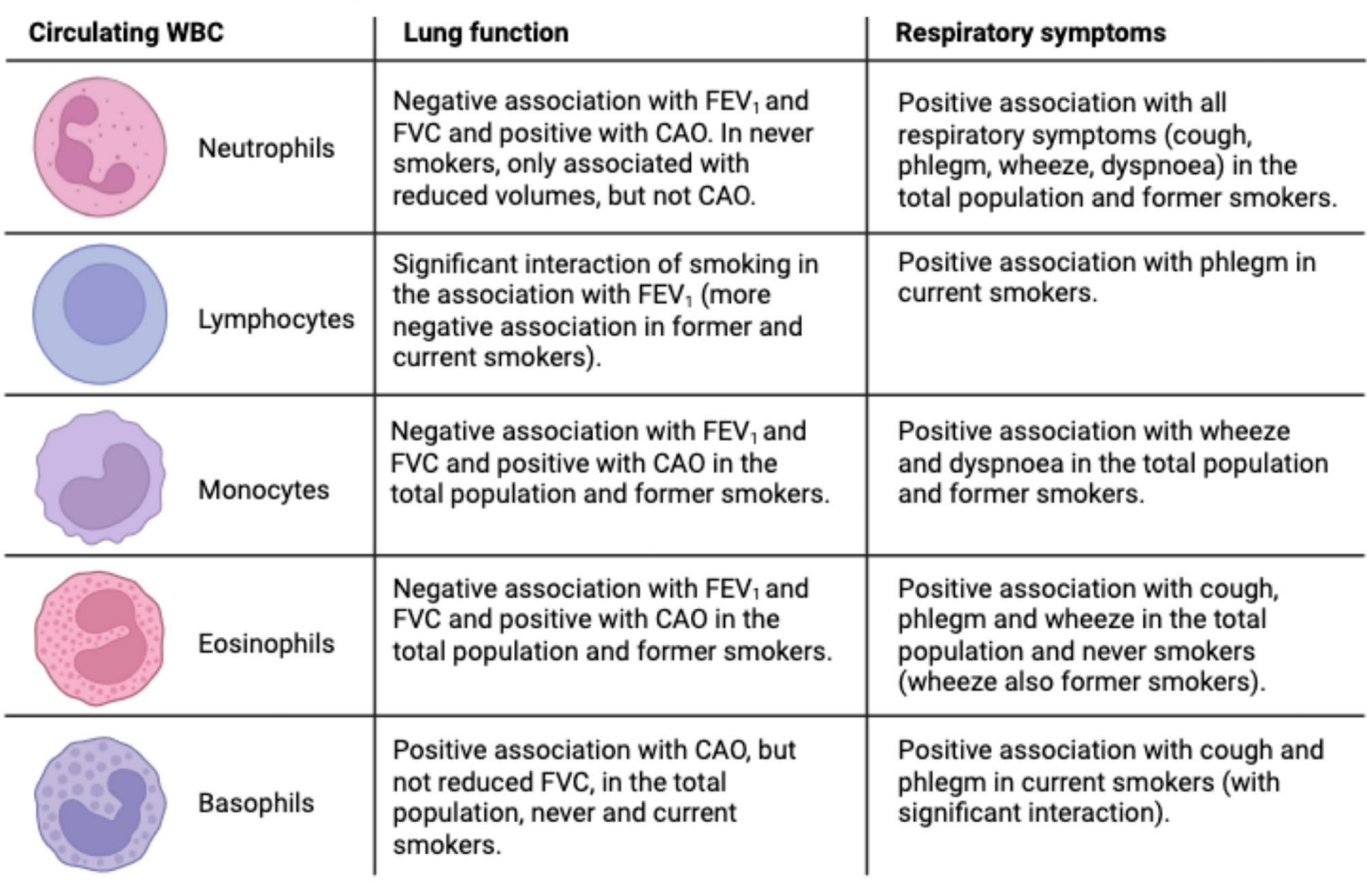
Summary of the findings of the study: associations between inflammatory markers and outcomes (lung function, respiratory symptoms), stratified by smoking status. Image created with BioRender.com. Abbreviations: WBC = white blood cells, FEV_1_ = forced expiratory volume in the 1 st second, FVC = forced vital capacity, CAO = chronic airflow obstruction, defined as post-bronchodilatory FEV_1_/FVC values below 5th percentile.

In this study, we found significant associations between circulating markers of systemic inflammation, lung function and respiratory symptoms in a general population cohort from Northern Europe, with different patterns according to smoking status. The main findings are summarized in Fig. 3.

Elevated blood neutrophils were associated with all respiratory symptoms (cough and phlegm – both without a cold and chronic –, wheeze, and dyspnoea), reduction of all lung function indices (FEV_1_, FVC, FEV_1_/FVC) and presence of CAO. This is in keeping with the established role of neutrophilic inflammation in the pathogenesis of obstructive lung disease, primarily COPD^27^, but also subtypes of asthma^9^. Lewis et al.^11^ also found an association between blood neutrophil levels and persistent cough, phlegm, dyspnoea, and reduced FEV_1_, but not wheeze, in a British general population sample. Nerpin et al.^5^ found an inverse relationship between blood neutrophils and pre-bronchodilatory FEV_1_, FVC, FEV_1_/FVC in the NHANES 2007–08 and 2009–10 surveys. When stratified by smoking status, the associations of neutrophils with reduced FEV_1_ and FVC – but not airflow obstruction – persisted in never-smokers. This suggests that neutrophilic inflammation can be related to lung restriction independently of cigarette smoking. Restrictive spirometric patterns are known to be related to metabolic syndrome, diabetes and obesity, with evidence that the reduction in lung function may precede the other manifestations^28^. The pathogenetic link is thought to be systemic inflammation, although the exact mechanisms are unknown^28^. In metabolic diseases, neutrophils release enzymes like myeloperoxidase and neutrophil elastase which promote insulin resistance and inflammation^29^. It is possible that these mechanisms are also implicated in the pathogenesis of low lung function.

Elevated blood eosinophils were associated with cough and phlegm in the absence of a cold, wheeze, reduced FEV_1_, reduced FVC, and presence of CAO. These findings also align with Lewis et al.^11^, although their study also evidenced an association with persistent cough. The association between eosinophils and wheeze in our study was present both in never and former smokers, while the association with reduced lung function was found only in former smokers, and the one with cough and phlegm only in never smokers. This might reflect the multiple aetiologies behind the findings, such as asthma, COPD, their overlap, infections, and potentially other conditions. It is well established that eosinophilia is related to type-2 inflammation, with eosinophils produced in response to IL-5 and further regulated in response to IL-4 and IL-13^30^. Type-2 inflammation is a feature of asthma and of a subset of subjects with COPD, although this role is more controversial^31^. There can be multiple differences in the nature of type-2 inflammation between asthma and COPD, such as the type of inflammatory mediators, the role of IgE in mast cell activation and the pathophysiology of mucus hypersecretion^32^. In our study, it is possible that the association between eosinophils and reduced lung function in former smokers might reflect type-2 inflammation in COPD, while the association with cough and phlegm in never smokers might reflect type-2 inflammation in asthma.

We also evidenced the association of circulating basophils with wheeze, dyspnoea, CAO and reduced FEV_1_, but not FVC. This might suggest that basophils are involved in airflow obstruction, but not restriction. The association with CAO was present both in never and current smokers, although the significance of the latter was affected by the small sample size. In current smokers, basophils were also related to cough and phlegm. Lewis et al.^11^ also evidenced significant associations between circulating basophils and wheeze, dyspnoea, and reduced FEV_1_, but not cough and phlegm, although their study did not stratify for smoking status. Only a few studies have explored the role of basophils in COPD and asthma. Winter et al. found that a mast cell/basophil gene signature measured in sputum was associated with eosinophilic inflammation and lower lung function in severe asthma^33^ and COPD^34^. Jogdand et al.^35^ described both eosinophils and basophils in histological lung COPD samples, with tissue density that increased with disease severity. Mast cells and basophils originate from common precursors. Recent studies hypothesized that mast cells could be a link between COPD and asthma^36,37^. It is possible that basophils may be related to airflow obstruction in never smokers in our study with IgE-dependent mechanisms typical of asthma^32^, while they may be related to airflow obstruction and mucus hypersecretion in current smokers with IgE-independent mechanisms typical of COPD^38^.

We found associations of circulating monocytes with wheeze, dyspnoea, reduced FEV_1_, reduced FVC and CAO. In stratified analyses, the association with reduced lung function was more evident in former smokers. Similarly, Halper-Stromberg et al.^39^ reported cross-sectional and longitudinal associations of monocytes with reduced FEV_1_ in the COPDGene and ECLIPSE cohorts, which consist of former and current smokers. Other studies confirmed associations of increased monocytes with reduced lung function in population-based samples^11,12^ and also reported associations with persistent cough and phlegm^11^. Blood monocytes can be recruited into the lung and differentiate into alveolar macrophages, key cells in COPD pathogenesis, which are further activated by cigarette smoking amplifying the inflammatory process^40^.

We did not find significant associations between blood lymphocyte counts and lung function in the total population, which is in line with the study of Lewis et al.^11^. However, we found a significant association between increased lymphocytes and phlegm in current smokers, confirmed by a significant interaction. Smoking status also modified the association between lymphocytes and FEV_1_, with more severe lung function impairment in former and current smokers. This might support a role for increased blood lymphocytes in COPD pathogenesis, although it has also been reported lower cross-sectional FEV_1_ and greater FEV_1_ decline in current and former smokers with baseline lymphopenia^39^. One potential explanation for this difference could be the severity of the disease, as the mean FEV_1_ in the COPDGene and ECLIPSE cohorts^39^ was much lower than in our study. Our study also examined the NLR, an index that can reflect inflammation associated with neutrophilia and impaired immune response associated with reduced lymphocytes^10,26,41^. The NLR has been proposed as a marker of activity and severity of COPD and a predictor of COPD exacerbations. In our study, the NLR showed a profile of association with lung function and respiratory symptoms substantially in line with that of neutrophils.

At present, only blood eosinophils are used as a biomarker of respiratory disease in clinical practice, specifically in the diagnosis and management of COPD^42^ and asthma^43^. Our study confirmed their relationship with reduced lung function and presence of respiratory symptoms (cough, phlegm, wheeze). In addition, our study showed a significant association between circulating basophils, lung function and respiratory symptoms, which to our knowledge has been described before only in a British population cohort^11^. In particular, they were related to airflow obstruction in the whole population, and to cough and phlegm in current smokers. We hypothesize that they might be helpful in differentiating, together with eosinophils, type-2 inflammation in asthma and COPD based on IgE-dependent and Ig-E-independent mechanisms, respectively. This possibility should be confirmed in future studies with other demographics and geographical settings, as well as mechanistic studies.

Our study also suggested that different markers of systemic inflammation may be related to different types of lung function impairment. We observed that circulating neutrophils were related to both airflow obstruction and reduced lung volumes (FEV_1_ and FVC) in the total population and in former smokers. In never-smokers, they were related to reduced FEV_1_ and FVC, but not CAO. Accordingly, they could reflect both obstructive and restrictive respiratory disease. The hypothesis of neutrophilic systemic inflammation linked to lung restriction would need to be confirmed in future studies using static lung volumes to assess restrictive impairment. Blood eosinophils and basophils, on the other hand, appeared to be related to CAO and reduced FEV_1_ to a larger extent than FVC, supporting a predominant role in airflow obstruction rather than restriction.

The strengths of our study include the general population sample, the use of standardized protocol and questionnaires across the study sites and the conduction of spirometry by trained and certified technicians. In addition, all spirometry curves were visually inspected centrally, and only those that passed quality control were used in this study. Compared to previous studies, our study investigated a larger number of outcomes (multiple lung function variables and respiratory symptoms) and it was multi-center, which increases generalizability. Our study also has limitations, including self-reporting respiratory symptoms, which may be affected by recall bias, and the fact that the mean age of our population was 68 years, which impedes the extrapolation of our findings to younger age groups. However, the demographics of the population may provide unique insights on the relationship between systemic inflammation and respiratory health in older adults on whom there is little data. The cross-sectional design does not allow us to infer causality; longitudinal studies are needed to establish temporal relationships between the variables involved. We did not have allergic sensitisation data on the participants, which would have been useful to consider in the current study. The proportion of current smokers in this population was low and this might have affected the significancy of the results. We focused on certain markers of systemic inflammation because we were interested in the individual contribution of WBC sub-populations, but other markers such as the systemic immune inflammation index (SII), the systemic inflammation response index (SIRI), or the aggregate index of systemic inflammation (AISI) may be worth exploring in future studies^44^.

In conclusion, larger counts of different WBC sub-populations are associated with different lung function indices and respiratory symptoms in the general population. Novel findings of our study include an association of blood basophils with CAO in the general population and with cough and phlegm in current smokers. Together with eosinophils, they might be useful in differentiating the type of inflammation in asthma and COPD. Blood neutrophils were related to reduced FEV_1_ and reduced FVC – but not CAO – in never smokers, suggesting a potential pathogenetic role in restrictive lung disease. These findings warrant further investigation in future studies.

## Supplementary Information

Below is the link to the electronic supplementary material.

Supplementary Material 1

## Data Availability

Data can be available on a collaborative basis upon reasonable request. Data requests can be directed to Dr. Christer Janson (christer.janson@medsci.uu.se).
