# Supplementary material for: The association between systemic inflammation, lung function and respiratory symptoms in the BOLD study in Northern Europe
Source: Sci Rep. 2025 Aug 22;15:30929. doi: 10.1038/s41598-025-16776-x (PMC12373827; doi:10.1038/s41598-025-16776-x)
Supplement: Supplementary file 1 — Supplementary Material 1 [file 41598_2025_16776_MOESM1_ESM.pdf]

Supplementary Data

The association between systemic inflammation, lung function and respiratory symptoms in the BOLD study in Northern Europe

Lucia Cestelli, Andre F.S. Amaral, Bryndis Benediktsdottir, Thorarinn Gislason, Rain Jõgi, Andrei Malinovschi, Rune Nielsen, Christer Janson

Table of contents

**Table S1**	Number of participants in the BOLD baseline and follow-up study	p. 2
**Table S2**	Characteristics of the participants (never smokers)	p. 3
**Table S3**	Characteristics of the participants (former smokers)	p. 4
**Table S4**	Characteristics of the participants (current smokers)	p. 5
**Table S5**	Interaction of smoking status in the association between inflammatory markers and lung function	p. 6
**Table S6**	Interaction of smoking status in the association between inflammatory markers and respiratory symptoms	p. 7
**Table S7**	Unadjusted associations between inflammatory markers and post-bronchodilatory lung function	p. 8
**Table S8**	Unadjusted associations between inflammatory markers and respiratory symptoms	p. 9
**Figure S1**	Meta-analysis inflammatory markers and FEV_1 (part 1)	p. 10
**Figure S2**	Meta-analysis inflammatory markers and FEV_1 (part 2)	p. 11
**Figure S3**	Meta-analysis inflammatory markers and FVC (part 1)	p. 12
**Figure S4**	Meta-analysis inflammatory markers and FVC (part 2)	p. 13
**Figure S5**	Meta-analysis inflammatory markers and FEV_1 /FVC(part 1)	p. 14
**Figure S6**	Meta-analysis inflammatory markers and FEV_1/FVC (part 2)	p. 15
**Figure S7**	Meta-analysis inflammatory markers and CAO (part 1)	p. 16
**Figure S8**	Meta-analysis inflammatory markers and CAO (part 2)	p. 17

Table S1. Number of invited people, ineligible people, non-responders and responders in the Northern European Burden of Obstructive Lung Disease (BOLD) sites at baseline and follow-up.

	Reykjavik (Iceland)	Bergen (Norway)	Uppsala (Sweden)	Tartu (Estonia)	Total
**Baseline (2004–2010)**					
Invited	1000	1130	998	1440	4568
Ineligible	94	133	31	482	740
Non-responders	146	290	379	300	1115
Responders	760	707	588	658	2713
**Follow-up (2019–2021)**					
Invited	757	661	551	619	2588
Ineligible (dead)	260 (187)	198 (139)	106 (52)	219 (219)	783 (597)
Non-responders	119	165	170	1	455
Responders	378	298	275	399	1350
Responders with available WBC count	371	296	266	305	1238

WBC= white blood cell

Table S2. Characteristics of the participants (never smokers, n=533) in the four North European sites of the Burden of Obstructive Lung Disease follow-up study.

Demographics/Anthropometrics					
	All<br>n=533	Reykjavik<br>n=149	Bergen<br>n=103	Uppsala<br>n=112	Tartu<br>n=169
Age (years)	68.6±9.4	67.2±9.5	70.5±8.9	66.8±8.9	69.9±9.8
Women	52.5	45.0	46.6	50.0	64.5
BMI (kg/m^2)					
<18.5	0.2	0.0	0.0	0.0	0.6
18.5–24.9	27.5	16.8	43.7	33.9	23.1
25–29.9	42.3	45.0	39.8	48.6	37.3
≥30	30.0	38.3	16.5	17.4	39.0
Blood inflammatory markers					
	n=533	n=149	n=103	n=112	n=169
Total WBC (10^9/L)	5.9±2.5	6.0±3.2	5.7±3.3	6.1±2.1	5.8±1.5
Neutrophils (10^9/L)	3.3±1.1	3.2±1.0	3.0±1.0	3.5±1.1	3.4±1.1
Lymphocytes (10^9/L)	1.9±2.1	2.1±2.9	2.0±2.8	1.8±1.3	1.7±0.5
NLR	2.0±0.9	1.9±0.8	1.9±1.0	2.2±0.9	2.1±0.9
Monocytes (10^9/L)	0.50±0.15	0.50±0.15	0.48±0.14	0.52±0.16	0.49±0.15
Eosinophils (10^9/L)	0.14(0.1)	0.2(0.1)	0.1(0.1)	0.13(0.14)	0.13(0.11)
Basophils (10^9/L)	0.03(0.06)	0.0(0.1)	0.0(0.1)	0.04(0.03)	0.03(0.03)
Post-bronchodilatory lung function					
	n=451	n=138	n=98	n=98	n=117
FEV_1 (%pred)	98.6±15.6	95.8±14.9	99.7±14.1	95.8±15.5	103.2±16.6
FVC (%pred)	97.0±14.3	95.5±14.3	97.2±12.6	93.4±13.9	101.5±14.9
FEV_1/FVC (%pred)	101.5±8.0	100.2±8.3	102.6±8.4	102.3±6.7	101.4±8.3
CAO	5.8	8.0	5.1	1.0	7.7
Respiratory symptoms					
	n=533	n=149	n=103	n=112	n=169
Cough w/o cold	25.1	35.6	16.5	24.1	21.9
Chronic cough	8.4	13.4	5.8	8.9	5.3
Phlegm w/o cold	12.6	11.4	10.7	16.1	12.4
Chronic phlegm	6.7	9.4	4.8	8.9	4.1
Wheeze	15.4	16.8	12.6	17.0	14.8
mMRC≥2	6.1	7.2	3.2	8.7	5.0

Data are presented as mean±SD, median (IQR) or %, as appropriate. BMI= body mass index, WBC = white blood cells, NLR= neutrophil-lymphocyte ratio, FEV_1= forced expiratory volume in the 1st second, FVC= forced vital capacity, CAO= chronic airflow obstruction, mMRC= modified Medical Research Council dyspnea scale.

Table S3. Characteristics of the participants (former smokers, n=548) in the four North European sites of the Burden of Obstructive Lung Disease follow-up study.

Demographics/Anthropometrics					
	All<br>n=548	Reykjavik<br>n=182	Bergen<br>n=147	Uppsala<br>n=127	Tartu<br>n=92
Age (years)	68.5±8.5	66.7±7.6	70.4±9.0	69.5±7.9	67.6±9.6
Women	42.7	46.7	45.6	38.6	35.9
BMI (kg/m^2)					
<18.5	0.2	0.6	0.0	0.0	0.0
18.5–24.9	24.9	19.2	37.4	29.4	9.8
25–29.9	42.4	41.2	42.9	45.2	40.2
≥30	32.5	39.0	19.7	25.4	50.0
Blood inflammatory markers					
	n=548	n=182	n=147	n=127	n=92
Total WBC (10^9/L)	6.1±1.7	5.7±1.4	6.0±1.6	6.5±2.2	6.2±1.5
Neutrophils (10^9/L)	3.4±1.2	3.1±1.0	3.4±1.3	3.8±1.4	3.5±1.2
Lymphocytes (10^9/L)	1.9±0.9	1.8±0.6	1.8±0.6	1.9±0.4	1.9±0.6
NLR	2.0±1.1	1.8±0.8	2.0±1.0	2.3±1.2	2.1±1.2
Monocytes (10^9/L)	0.54±0.17	0.51±0.15	0.54±0.18	0.57±0.18	0.55±0.15
Eosinophils (10^9/L)	0.2(0.17)	0.2(0.2)	0.2(0.2)	0.15(0.12)	0.16(0.14)
Basophils (10^9/L)	0.04(0.1)	0.0(0.1)	0.0(0.1)	0.04(0.03)	0.04(0.03)
Post-bronchodilatory lung function					
	n=468	n=161	n=134	n=115	n=58
FEV_1 (%pred)	94.0±16.4	90.6±15.2	96.5±16.0	94.8±17.4	96.2±17.7
FVC (%pred)	95.5±13.5	93.5±12.8	98.0±12.8	93.9±14.6	98.7±13.1
FEV_1/FVC (%pred)	98.0±10.1	96.7±10.7	97.9±9.8	100.5±8.2	96.9±11.9
CAO	10.3	11.8	10.4	6.1	13.8
Respiratory symptoms					
	n=405	n=120	n=116	n=93	n=76
Cough w/o cold	26.1	34.1	21.1	26.8	17.4
Chronic cough	9.7	13.2	7.5	6.4	10.9
Phlegm w/o cold	20.1	22.5	25.2	15.7	18.5
Chronic phlegm	11.3	12.1	12.9	8.7	10.9
Wheeze	21.2	27.5	16.3	18.1	20.6
mMRC≥2	5.6	5.7	2.3	6.6	10.0

Data are presented as mean±SD, median (IQR) or %, as appropriate. BMI= body mass index, WBC = white blood cells, NLR= neutrophil-lymphocyte ratio, FEV_1= forced expiratory volume in the 1st second, FVC= forced vital capacity, CAO= chronic airflow obstruction, mMRC= modified Medical Research Council dyspnea scale.

Table S4. Characteristics of the participants (current smokers, n=148) in the four North European sites of the Burden of Obstructive Lung Disease follow-up study.

Demographics/Anthropometrics					
	All n=148	Reykjavik n=40	Bergen n=46	Uppsala n=18	Tartu n=44
Age (years)	64.9±7.2	65.4±7.0	65.1±6.3	65.6±7.5	63.9±8.3
Women	43.2	42.5	50.0	33.3	40.9
BMI (kg/m^2)					
<18.5	1.3	0.0	0.0	0.0	4.5
18.5–24.9	31.8	27.5	30.4	50.0	29.6
25–29.9	41.9	47.5	45.6	22.2	40.9
≥30	25.0	25.0	23.9	27.8	25.0
Blood inflammatory markers					
	n=148	n=40	n=46	n=18	n=44
Total WBC (10^9/L)	7.0±1.7	6.5±1.6	7.0±1.6	7.4±2.0	7.3±1.6
Neutrophils (10^9/L)	4.0±1.3	3.4±1.0	4.0±1.3	4.4±1.4	4.3±1.4
Lymphocytes (10^9/L)	2.2±0.6	2.2±0.7	2.1±0.6	2.1±0.5	2.1±0.6
NLR	2.0±0.9	1.7±1.0	2.0±0.9	2.1±0.6	2.2±0.9
Monocytes (10^9/L)	0.58±0.17	0.59±0.17	0.57±0.18	0.61±0.21	0.59±0.14
Eosinophils (10^9/L)	0.2(0.2)	0.2(0.2)	0.2(0.2)	0.16(0.14)	0.18(0.12)
Basophils (10^9/L)	0.06(0.1)	0.05(0.1)	0.1(0.1)	0.05(0.04)	0.05(0.04)
Post-bronchodilatory lung function					
	n=128	n=38	n=44	n=17	n=29
FEV_1 (%pred)	87.6±15.6	84.6±17.8	86.5±15.8	91.3±11.8	91.1±13.7
FVC (%pred)	94.2±15.7	92.3±15.8	91.9±17.8	95.0±13.2	99.7±12.5
FEV_1/FVC (%pred)	93.1±12.0	91.2±14.2	94.5±11.8	96.6±11.1	91.4±9.1
CAO	25.8	31.6	22.7	17.6	27.6
Respiratory symptoms					
	n=148	n=40	n=46	n=18	n=44
Cough w/o cold	35.1	35.0	37.0	44.4	29.5
Chronic cough	8.3	10.3	6.5	12.5	6.8
Phlegm w/o cold	28.4	20.0	30.4	38.9	29.5
Chronic phlegm	11.0	12.5	15.2	0.0	9.1
Wheeze	38.5	37.5	45.6	33.3	34.1
mMRC≥2	6.7	10.8	2.2	11.8	5.9

Data are presented as mean±SD, median (IQR) or %, as appropriate. BMI= body mass index, WBC = white blood cells, NLR= neutrophil-lymphocyte ratio, FEV_1= forced expiratory volume in the 1st second, FVC= forced vital capacity, CAO= chronic airflow obstruction, mMRC= modified Medical Research Council dyspnea scale.

Table S5. Interactions between smoking status and inflammatory markers in relation to the association with post-bronchodilatory lung function (β coefficients and odds ratios (OR) per unit with 95% confidence intervals (CI) adjusted for gender, age, body mass index and study site).

	FEV_1 %pred		FVC %pred	
**Smoking status**	Coef. (95% CI)	p value	Coef. (95% CI)	p value
Total WBC (10^9/L)				
Former	**-2.06 (-3.11, -1.02)**	**<0.001**	**-1.15 (-2.05, -0.25)**	**0.012**
Current	-1.59 (-3.29, 0.11)	0.067	-0.90 (-2.37, 0.56)	0.226
Neutrophils (10^9/L)				
Former	-0.36 (-2.13, 1.41)	0.690	0.42 (-1.10, 1.94)	0.586
Current	0.27 (-2.19, 2.73)	0.830	0.83 (-1.29, 2.94)	0.442
Lymphocytes (10^9/L)				
Former	**-2.60 (-5.13, -0.08)**	**0.044**	-1.84 (-4.00, 0.33)	0.096
Current	-4.21 (-8.89, 0.48)	0.079	-3.12 (-7.13, 0.90)	0.128
NLR				
Former	0.82 (1.32, 2.96)	0.452	0.76 (-1.07, 2.59)	0.413
Current	1.21 (-2.22, 4.64)	0.489	0.67 (-2.28, 3.61)	0.657
Monocytes (10^{10}/L)				
Former	-1.00 (-2.28, 0.28)	0.127	-0.55 (-1.65, 0.56)	0.334
Current	-0.10 (-1.95, 1.75)	0.913	0.01 (-1.59, 1.61)	0.989
Eosinophils (10^{10}/L)				
Former	**-1.51 (-2.93, -0.09)**	**0.038**	-0.70 (-1.92, 0.53)	0.264
Current	-0.81 (-3.33, 1.70)	0.527	-1.43 (-3.59, 0.73)	0.193
Basophils (10^{10}/L)				
Former	4.03 (-0.67, 8.72)	0.093	3.16 (-0.86, 7.19)	0.123
Current	-1.09 (-7.96, 5.78)	0.755	-0.68 (6.57, 5.20)	0.820
	**FEV_1/FVC %pred**		**CAO**	
	Coef. (95% CI)	p value	OR (95% CI)	p value
Total WBC (10^9/L)				
Former	**-1.10 (-1.73, -0.47)**	**0.001**	**1.29 (1.03, 1.63)**	**0.030**
Current	-0.69 (-1.71, 0.34)	0.188	1.17 (0.87, 1.56)	0.307
Neutrophils (10^9/L)				
Former	-1.04 (-2.10, 0.03)	0.057	1.19 (0.78, 1.83)	0.413
Current	0.46 (-1.94, 1.03)	0.547	1.02 (0.63, 1.67)	0.922
Lymphocytes (10^9/L)				
Former	-0.80 (-2.33, 0.72)	0.300	1.36 (0.54, 3.40)	0.517
Current	-1.69 (-4.51, 1.13)	0.240	1.77 (0.64, 4.88)	0.272
NLR				
Former	-0.12 (-1.41, 1.16)	0.851	1.05 (0.65, 1.68)	0.851
Current	0.86 (-1.20, 2.93)	0.413	0.81 (0.44, 1.48)	0.495
Monocytes (10^{10}/L)				
Former	-0.57 (-1.35, 0.20)	0.147	1.06 (0.78, 1.43)	0.724
Current	-0.05 (-1.16, 1.07)	0.935	0.98 (0.69, 1.39)	0.912
Eosinophils (10^{10}/L)				
Former	-0.80 (-1.65, 0.06)	0.068	1.25 (0.93, 1.66)	0.134
Current	0.67 (-0.85, 2.19)	0.387	0.96 (0.63, 1.46)	0.846
Basophils (10^{10}/L)				
Former	0.79 (-2.03, 3.60)	0.583	0.71 (0.27, 1.90)	0.497
Current	-1.17 (-5.29, 2.95)	0.577	0.86 (0.27, 2.78)	0.799

Never smokers is the reference category. FEV_1= forced expiratory volume in the 1st second, FVC= forced vital capacity, CAO= chronic airflow obstruction, WBC = white blood cells, NLR= neutrophil-lymphocyte ratio. Interactions with p value < 0.05 are in bold.

Table S6. Interactions between smoking status and inflammatory markers in relation to the association with respiratory symptoms (odds ratios (OR) per unit with 95% confidence intervals (CI) adjusted for gender, age, body mass index and study site).

	**Cough w/o cold**		**Chronic cough**		**Phlegm w/o cold**	
**Smoking status**	OR (95% CI)	p value	OR (95% CI)	p value	OR (95% CI)	p value
Total WBC (10^9/L)						
Former	1.07 (0.94, 1.23)	0.309	1.10 (0.93, 1.31)	0.274	1.12 (0.96, 1.31)	0.139
Current	**1.40 (1.10, 1.77)**	**0.005**	0.99 (0.69, 1.44)	0.967	**1.28 (1.00, 1.64)**	**0.046**
Neutrophils (10^9/L)						
Former	1.09 (0.86, 1.39)	0.484	1.18 (0.83, 1.67)	0.352	1.23 (0.92, 1.64)	0.160
Current	1.37 (0.98, 1.90)	0.063	0.83 (0.47, 1.45)	0.509	1.20 (0.83, 1.73)	0.335
Lymphocytes (10^9/L)						
Former	0.92 (0.68, 1.24)	0.578	0.97 (0.69, 1.36)	0.841	0.88 (0.61, 1.28)	0.501
Current	1.70 (0.93, 3.12)	0.084	1.86 (0.71, 4.84)	0.205	**2.05 (1.08, 3.91)**	**0.029**
NLR						
Former	1.07 (0.82, 1.41)	0.612	1.43 (0.90, 2.27)	0.131	1.32 (0.94, 1.85)	0.109
Current	1.24 (0.79, 1.93)	0.351	0.77 (0.28, 2.08)	0.600	1.17 (0.72, 1.90)	0.523
Monocytes (10^{10}/L)						
Former	1.02 (0.86, 1.22)	0.802	0.92 (0.71, 1.19)	0.509	0.92 (0.75, 1.13)	0.415
Current	1.20 (0.94, 1.53)	0.154	0.99 (0.67, 1.47)	0.962	0.97 (0.74, 1.27)	0.821
Eosinophils (10^{10}/L)						
Former	0.93 (0.77, 1.12)	0.443	1.09 (0.83, 1.44)	0.516	0.92 (0.75, 1.12)	0.386
Current	0.78 (0.57, 1.07)	0.119	0.77 (0.42, 1.41)	0.399	0.90 (0.65, 1.24)	0.518
Basophils (10^{10}/L)						
Former	0.86 (0.45, 1.63)	0.639	1.46 (0.53, 4.05)	0.463	1.82 (0.80, 4.15)	0.155
Current	2.18 (0.86, 5.51)	0.100	**4.99 (1.06, 23.6)**	**0.043**	**7.69 (2.43, 24.37)**	**0.001**
	**Chronic phlegm**		**Wheeze**		**mMRC≥2**	
	OR (95% CI)	p value	OR (95% CI)	p value	OR (95% CI)	p value
Total WBC (10^9/L)						
Former	1.13 (0.94, 1.36)	0.202	**1.26 (1.07, 1.50)**	**0.007**	1.12 (0.90, 1.40)	0.292
Current	0.99 (0.70, 1.40)	0.968	1.09 (0.86, 1.38)	0.465	1.02 (0.64, 1.63)	0.918
Neutrophils (10^9/L)						
Former	1.27 (0.87, 1.85)	0.208	**1.49 (1.12, 1.98)**	**0.007**	0.96 (0.62, 1.50)	0.869
Current	0.80 (0.46, 1.36)	0.406	1.21 (0.85, 1.71)	0.290	0.75 (0.38, 1.47)	0.402
Lymphocytes (10^9/L)						
Former	0.84 (0.52, 1.35)	0.469	1.08 (0.84, 1.39)	0.565	1.02 (0.59, 1.77)	0.934
Current	2.15 (0.89, 5.17)	0.087	0.97 (0.53, 1.74)	0.908	0.87 (0.25, 3.10)	0.836
NLR						
Former	1.57 (0.97, 2.53)	0.066	**1.52 (1.05, 2.19)**	**0.025**	0.94 (0.59, 1.50)	0.789
Current	0.87 (0.37, 2.03)	0.748	**1.67 (1.01, 2.74)**	**0.045**	1.06 (0.52, 2.17)	0.870
Monocytes (10^{10}/L)						
Former	0.88 (0.67, 1.16)	0.374	1.13 (0.93, 1.38)	0.217	1.10 (0.79, 1.53)	0.569
Current	0.87 (0.60, 1.27)	0.482	1.09 (0.84, 1.41)	0.499	1.12 (0.69, 1.83)	0.643
Eosinophils (10^{10}/L)						
Former	1.17 (0.86, 1.60)	0.311	1.16 (0.95, 1.42)	0.141	1.08 (0.73, 1.61)	0.708
Current	1.02 (0.62, 1.70)	0.930	1.06 (0.77, 1,47)	0.707	1.12 (0.60, 2.09)	0.721
Basophils (10^{10}/L)						
Former	**4.48 (1.41, 14.24)**	**0.011**	1.69 (0.81, 3.56)	0.165	1.18 (0.31, 4.49)	0.804
Current	**13.26 (2.62, 67.06)**	**0.002**	1.58 (0.60, 4.15)	0.352	2.57 (0.37, 17.91)	0.340

Never smokers is the reference category. mMRC= modified Medical Research Council dyspnea scale, WBC = white blood cells, NLR= neutrophil-lymphocyte ratio. Interactions with p value < 0.05 are in bold.

Table S7. Unadjusted associations between inflammatory markers and post-bronchodilatory lung function in the total population (β coefficients and odds ratios (OR) per unit with 95% confidence intervals (CI).

	**FEV_1 %pred**		**FVC %pred**	
	Coef. (95% CI)	p value	Coef. (95% CI)	p value
Total WBC (10^9/L)	-0.75 (-1.20, -0.29)	0.001	-0.59 (-0.98, -0.19)	0.004
Neutrophils (10^9/L)	-2.09 (-2.90, -1.27)	<0.001	-1.50 (-2.21, -0.80)	<0.001
Lymphocytes (10^9/L)	0.16 (-0.49, 0.81)	0.634	-0.01 (-0.56, 0.55)	0.975
NLR	-1.06 (-2.10, -0.02)	0.045	-0.86 (-1.76, 0.03)	0.059
Monocytes (10^{10}/L)	-1.76 (-2.37, -1.15)	<0.001	-1.05 (1-58, -0.52)	<0.001
Eosinophils (10^{10}/L)	-1.26 (-1.97, -0.56)	<0.001	-0.79 (-1.40, -0.18)	0.012
Basophils (10^{10}/L)	-2.17 (-4.44, 0.10)	0.061	0.06 (-1.90, 2.03)	0.951
	**FEV_1/FVC %pred**		**CAO**	
	Coef. (95% CI)	p value	OR (95% CI)	p value
Total WBC (10^9/L)	-0.22 (-0.49, 0.06)	0.125	1.07 (1.00, 1.15)	0.052
Neutrophils (10^9/L)	-0.71 (-1.21, -0.22)	0.005	1.27 (1.10, 1.47)	0.001
Lymphocytes (10^9/L)	0.14 (-0.25, 0.54)	0.470	0.97 (0.81, 1.16)	0.745
NLR	-0.27 (-0.89, 0.36)	0.402	1.19 (0.99, 1.43)	0.071
Monocytes (10^{10}/L)	-0.80 (-1.17, -0.43)	<0.001	1.26 (1.13, 1.41)	<0.001
Eosinophils (10^{10}/L)	-0.48 (-0.91, -0.05)	0.027	1.19 (1.05, 1.33)	0.004
Basophils (10^{10}/L)	-2.22 (-3.58, -0.85)	0.001	2.36 (1.55, 3.61)	<0.001

FEV_1= forced expiratory volume in the 1st second, FVC= forced vital capacity, CAO= chronic airflow obstruction, WBC = white blood cells, NLR= neutrophil-lymphocyte ratio.

Table S8. Unadjusted associations between inflammatory markers and respiratory symptoms in the total population (odds ratios (OR) per unit with 95% confidence intervals (CI).

	Cough w/o cold		Chronic cough		Phlegm w/o cold	
	OR (95% CI)	p value	OR (95% CI)	p value	OR (95% CI)	p value
Total WBC (10^9/L)	1.07 (1.01, 1.14)	0.025	1.06 (0.98, 1.13)	0.127	1.08 (1.02, 1.15)	0.014
Neutrophils (10^9/L)	1.20 (1.09, 1.33)	<0.001	1.11 (0.95, 1.29)	0.179	1.26 (1.13, 1.42)	<0.001
Lymphocytes (10^9/L)	1.00 (0.92, 1.09)	0.975	1.04 (0.95, 1.14)	0.354	0.99 (0.90, 1.10)	0.922
NLR	1.12 (0.99, 1.27)	0.072	0.93 (0.75, 1.15)	0.495	1.14 (0.99, 1.31)	0.062
Monocytes (10^{10}/L)	1.07 (1.00, 1.16)	0.065	1.06 (0.94, 1.19)	0.324	1.14 (1.05, 1.24)	0.003
Eosinophils (10^{10}/L)	1.15 (1.05, 1.25)	0.002	1.07 (0.94, 1.22)	0.283	1.18 (1.08, 1.30)	<0.001
Basophils (10^{10}/L)	1.28 (0.96, 1.71)	0.098	1.02 (0.64, 1.61)	0.942	1.40 (1.01, 1.95)	0.043
	**Chronic phlegm**		**Wheeze**		**mMRC≥2**	
	OR (95% CI)	p value	OR (95% CI)	p value	OR (95% CI)	p value
Total WBC (10^9/L)	1.05 (0.98, 1.13)	0.130	1.11 (1.04, 1.19)	0.003	1.10 (1.02, 1.18)	0.012
Neutrophils (10^9/L)	1.18 (1.02, 1.37)	0.024	1.24 (1.11, 1.38)	<0.001	1.49 (1.24, 1.78)	<0.001
Lymphocytes (10^9/L)	1.01 (0.90, 1.14)	0.880	1.04 (0.96, 1.12)	0.385	1.02 (0.89, 1.16)	0.789
NLR	1.07 (0.89, 1.28)	0.492	0.98 (0.85, 1.13)	0.828	1.39 (1.14, 1.70)	0.001
Monocytes (10^{10}/L)	1.10 (0.98, 1.23)	0.104	1.15 (1.06, 1.25)	0.001	1.24 (1.07, 1.44)	0.004
Eosinophils (10^{10}/L)	1.06 (0.94, 1.21)	0.349	1.26 (1.15, 1.38)	<0.001	1.02 (0.86, 1.22)	0.814
Basophils (10^{10}/L)	1.41 (0.92, 2.16)	0.118	1.84 (1.35, 2.52)	<0.001	1.66 (0.96, 2.85)	0.068

mMRC= modified Medical Research Council dyspnea scale, WBC = white blood cells, NLR= neutrophil-lymphocyte ratio.

Figure S1.

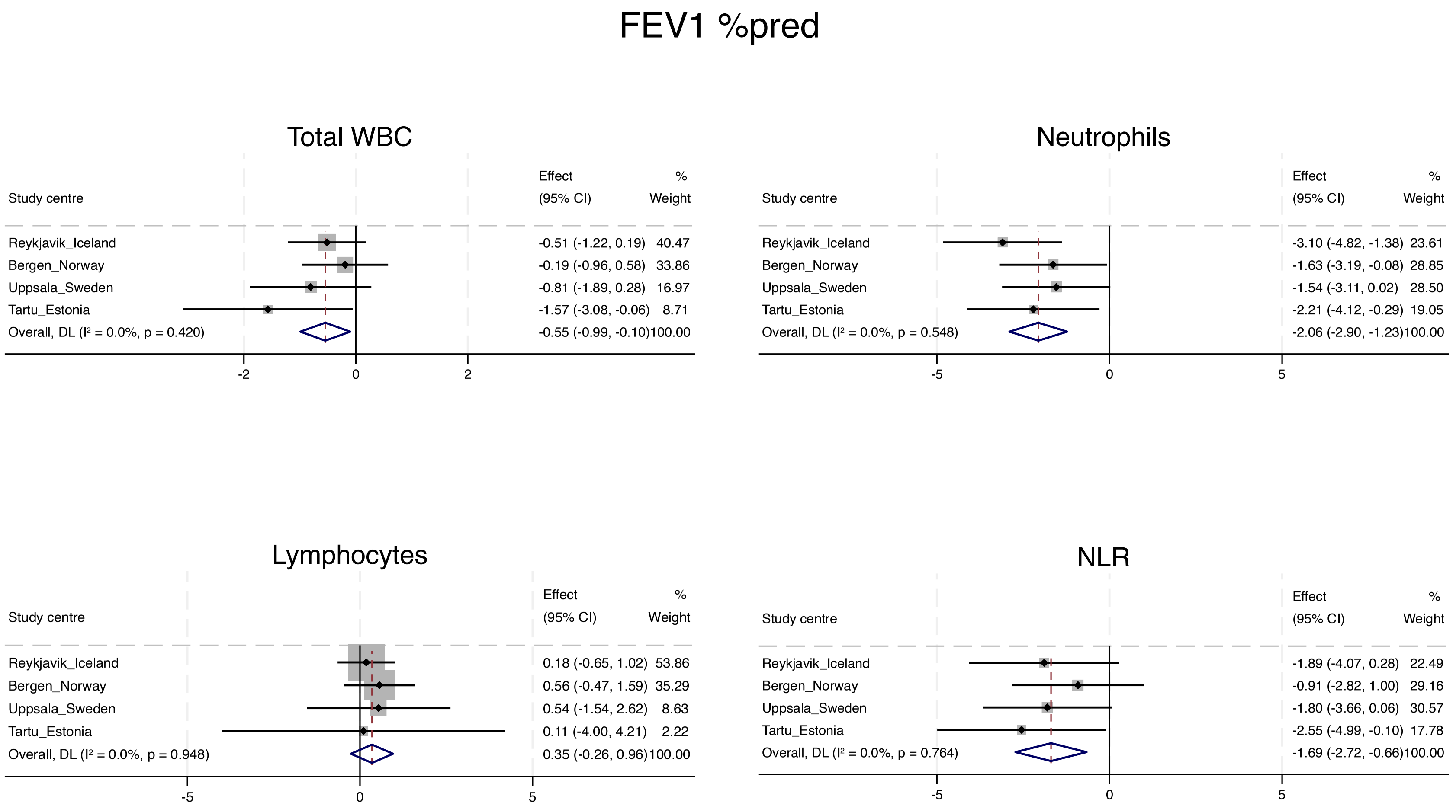
FEV1 %pred
Total WBC
Study centre
Effect
(95% CI)
%
Weight
Reykjavik_Iceland -0.51 (-1.22, 0.19) 40.47
Bergen_Norway -0.19 (-0.96, 0.58) 33.86
Uppsala_Sweden -0.81 (-1.89, 0.28) 16.97
Tartu_Estonia -1.57 (-3.08, -0.06) 8.71
Overall, DL (I² = 0.0%, p = 0.420) -0.55 (-0.99, -0.10)100.00
-2
0
2
Neutrophils
Study centre
Effect
(95% CI)
%
Weight
Reykjavik_Iceland -3.10 (-4.82, -1.38) 23.61
Bergen_Norway -1.63 (-3.19, -0.08) 28.85
Uppsala_Sweden -1.54 (-3.11, 0.02) 28.50
Tartu_Estonia -2.21 (-4.12, -0.29) 19.05
Overall, DL (I² = 0.0%, p = 0.548) -2.06 (-2.90, -1.23)100.00
-5
0
5
Lymphocytes
Study centre
Effect
(95% CI)
%
Weight
Reykjavik_Iceland 0.18 (-0.65, 1.02) 53.86
Bergen_Norway 0.56 (-0.47, 1.59) 35.29
Uppsala_Sweden 0.54 (-1.54, 2.62) 8.63
Tartu_Estonia 0.11 (-4.00, 4.21) 2.22
Overall, DL (I² = 0.0%, p = 0.948) 0.35 (-0.26, 0.96)100.00
-5
0
5
NLR
Study centre
Effect
(95% CI)
%
Weight
Reykjavik_Iceland -1.89 (-4.07, 0.28) 22.49
Bergen_Norway -0.91 (-2.82, 1.00) 29.16
Uppsala_Sweden -1.80 (-3.66, 0.06) 30.57
Tartu_Estonia -2.55 (-4.99, -0.10) 17.78
Overall, DL (I² = 0.0%, p = 0.764) -1.69 (-2.72, -0.66)100.00
-5
0
5

Figure S2.

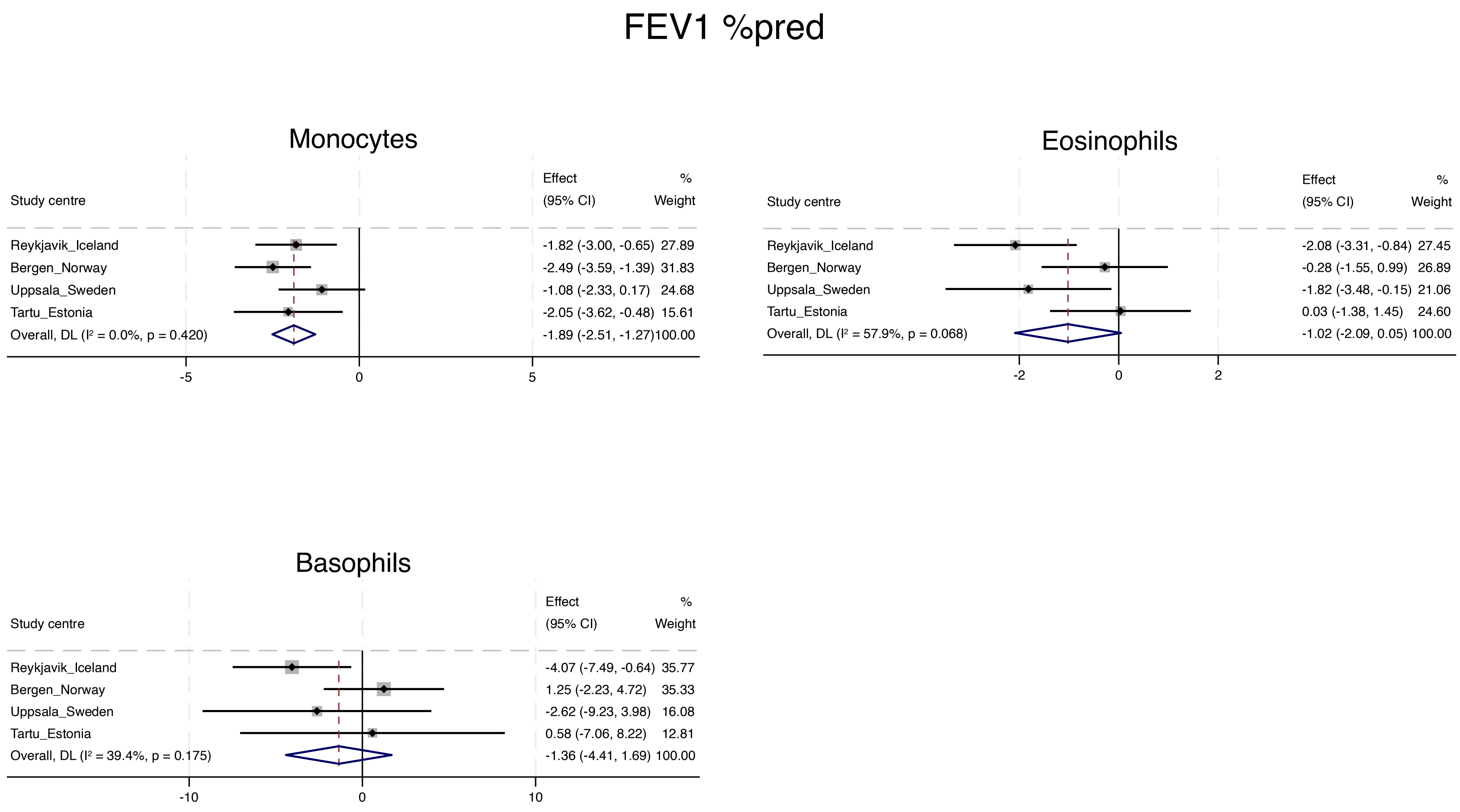
FEV1 %pred
Monocytes
Study centre
Effect (95% CI)
% Weight
Reykjavik_Iceland -1.82 (-3.00, -0.65) 27.89
Bergen_Norway -2.49 (-3.59, -1.39) 31.83
Uppsala_Sweden -1.08 (-2.33, 0.17) 24.68
Tartu_Estonia -2.05 (-3.62, -0.48) 15.61
Overall, DL (I² = 0.0%, p = 0.420) -1.89 (-2.51, -1.27)100.00
-5
0
5
Eosinophils
Study centre
Effect (95% CI)
% Weight
Reykjavik_Iceland -2.08 (-3.31, -0.84) 27.45
Bergen_Norway -0.28 (-1.55, 0.99) 26.89
Uppsala_Sweden -1.82 (-3.48, -0.15) 21.06
Tartu_Estonia 0.03 (-1.38, 1.45) 24.60
Overall, DL (I² = 57.9%, p = 0.068) -1.02 (-2.09, 0.05) 100.00
-2
0
2
Basophils
Study centre
Effect (95% CI)
% Weight
Reykjavik_Iceland -4.07 (-7.49, -0.64) 35.77
Bergen_Norway 1.25 (-2.23, 4.72) 35.33
Uppsala_Sweden -2.62 (-9.23, 3.98) 16.08
Tartu_Estonia 0.58 (-7.06, 8.22) 12.81
Overall, DL (I² = 39.4%, p = 0.175) -1.36 (-4.41, 1.69) 100.00
-10
0
10

Figure S3.

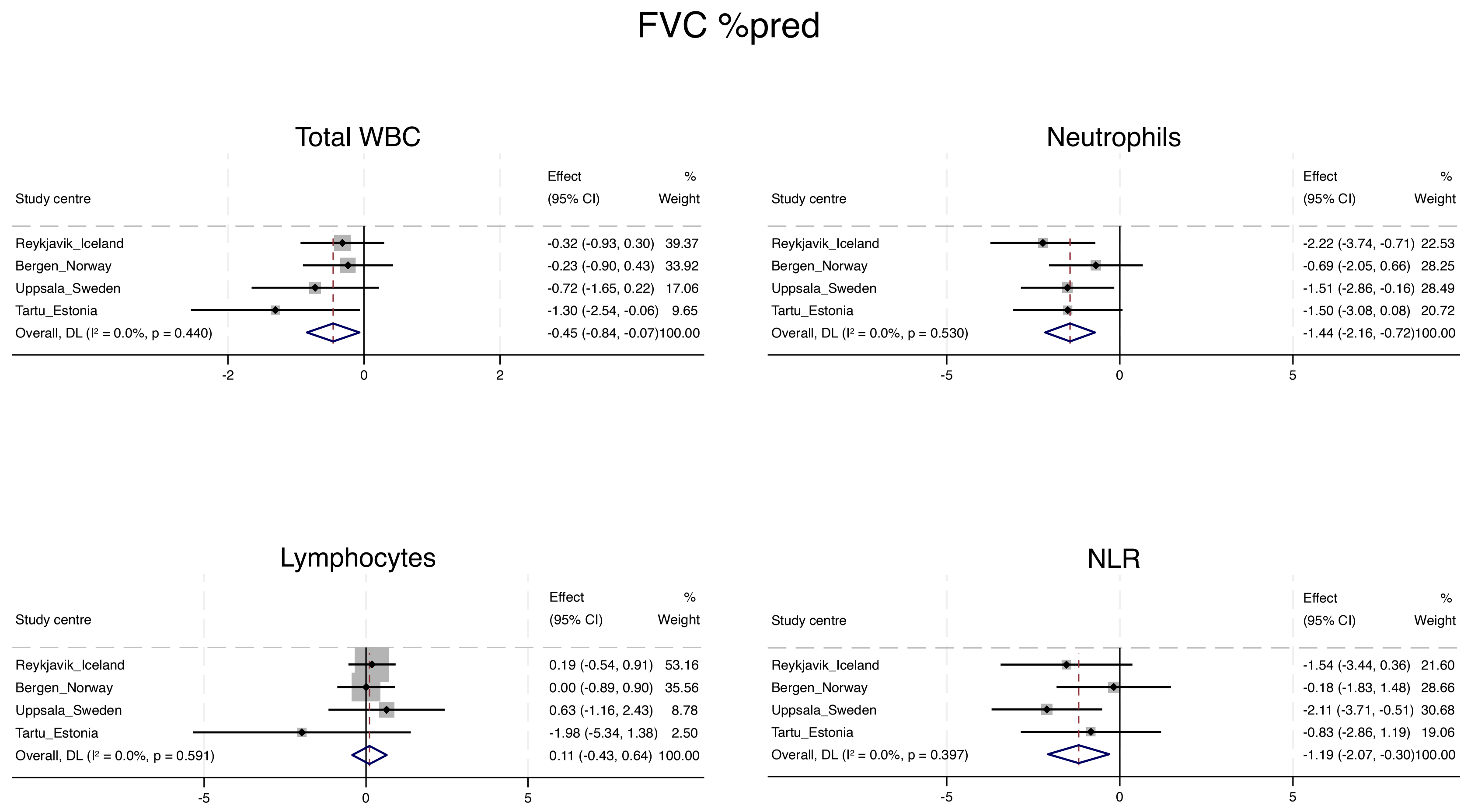
FVC %pred
Total WBC
Study centre
Effect (95% CI)
% Weight
Reykjavik_Iceland -0.32 (-0.93, 0.30) 39.37
Bergen_Norway -0.23 (-0.90, 0.43) 33.92
Uppsala_Sweden -0.72 (-1.65, 0.22) 17.06
Tartu_Estonia -1.30 (-2.54, -0.06) 9.65
Overall, DL (I² = 0.0%, p = 0.440) -0.45 (-0.84, -0.07) 100.00
-2
0
2
Neutrophils
Study centre
Effect (95% CI)
% Weight
Reykjavik_Iceland -2.22 (-3.74, -0.71) 22.53
Bergen_Norway -0.69 (-2.05, 0.66) 28.25
Uppsala_Sweden -1.51 (-2.86, -0.16) 28.49
Tartu_Estonia -1.50 (-3.08, 0.08) 20.72
Overall, DL (I² = 0.0%, p = 0.530) -1.44 (-2.16, -0.72) 100.00
-5
0
5
Lymphocytes
Study centre
Effect (95% CI)
% Weight
Reykjavik_Iceland 0.19 (-0.54, 0.91) 53.16
Bergen_Norway 0.00 (-0.89, 0.90) 35.56
Uppsala_Sweden 0.63 (-1.16, 2.43) 8.78
Tartu_Estonia -1.98 (-5.34, 1.38) 2.50
Overall, DL (I² = 0.0%, p = 0.591) 0.11 (-0.43, 0.64) 100.00
-5
0
5
NLR
Study centre
Effect (95% CI)
% Weight
Reykjavik_Iceland -1.54 (-3.44, 0.36) 21.60
Bergen_Norway -0.18 (-1.83, 1.48) 28.66
Uppsala_Sweden -2.11 (-3.71, -0.51) 30.68
Tartu_Estonia -0.83 (-2.86, 1.19) 19.06
Overall, DL (I² = 0.0%, p = 0.397) -1.19 (-2.07, -0.30) 100.00
-5
0
5

Figure S4.

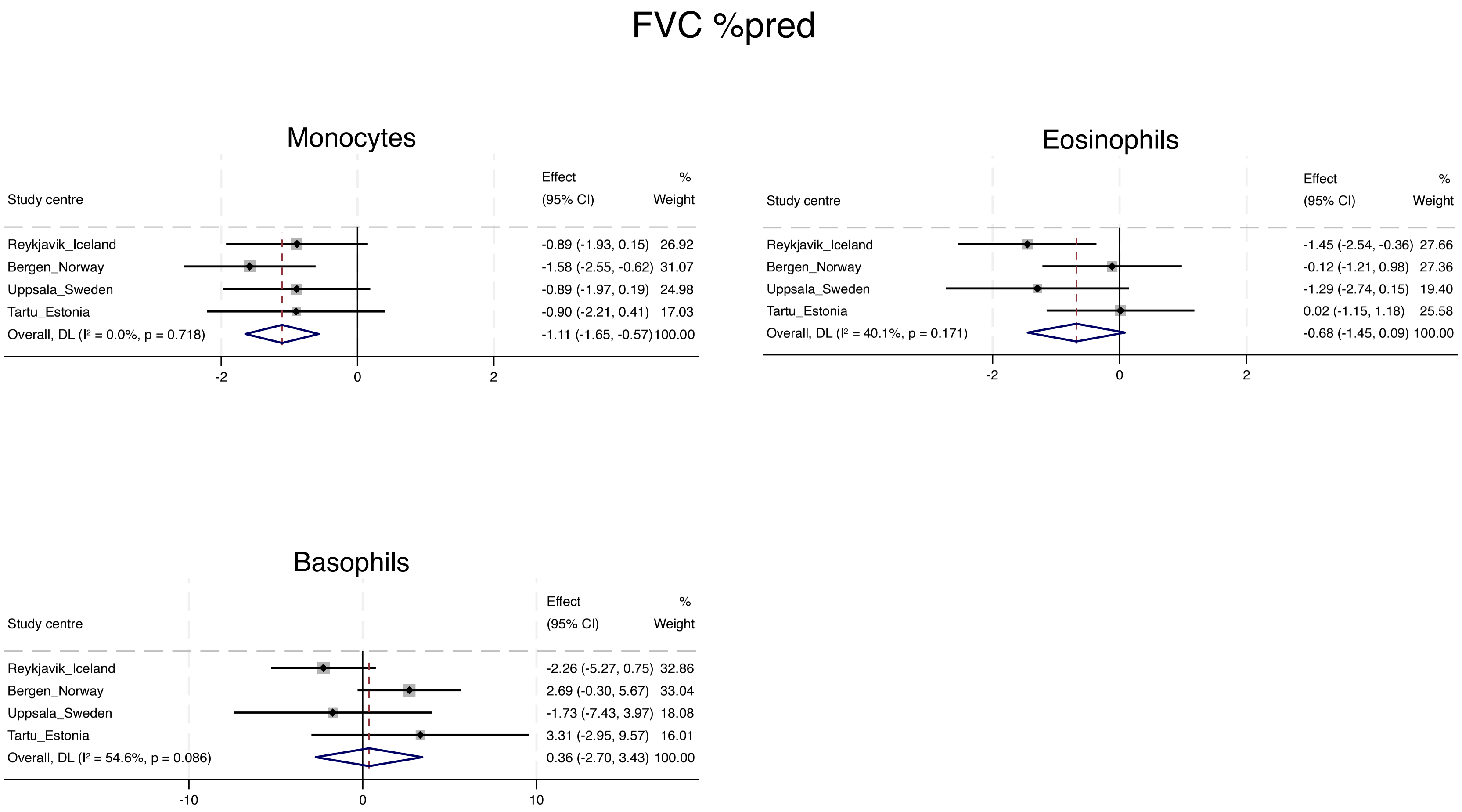

FVC %pred
Monocytes
Study centre
Effect (95% CI)
% Weight
Reykjavik_Iceland -0.89 (-1.93, 0.15) 26.92
Bergen_Norway -1.58 (-2.55, -0.62) 31.07
Uppsala_Sweden -0.89 (-1.97, 0.19) 24.98
Tartu_Estonia -0.90 (-2.21, 0.41) 17.03
Overall, DL (I² = 0.0%, p = 0.718) -1.11 (-1.65, -0.57) 100.00
-2
0
2
Eosinophils
Study centre
Effect (95% CI)
% Weight
Reykjavik_Iceland -1.45 (-2.54, -0.36) 27.66
Bergen_Norway -0.12 (-1.21, 0.98) 27.36
Uppsala_Sweden -1.29 (-2.74, 0.15) 19.40
Tartu_Estonia 0.02 (-1.15, 1.18) 25.58
Overall, DL (I² = 40.1%, p = 0.171) -0.68 (-1.45, 0.09) 100.00
-2
0
2
Basophils
Study centre
Effect (95% CI)
% Weight
Reykjavik_Iceland -2.26 (-5.27, 0.75) 32.86
Bergen_Norway 2.69 (-0.30, 5.67) 33.04
Uppsala_Sweden -1.73 (-7.43, 3.97) 18.08
Tartu_Estonia 3.31 (-2.95, 9.57) 16.01
Overall, DL (I² = 54.6%, p = 0.086) 0.36 (-2.70, 3.43) 100.00
-10
0
10

Figure S5.

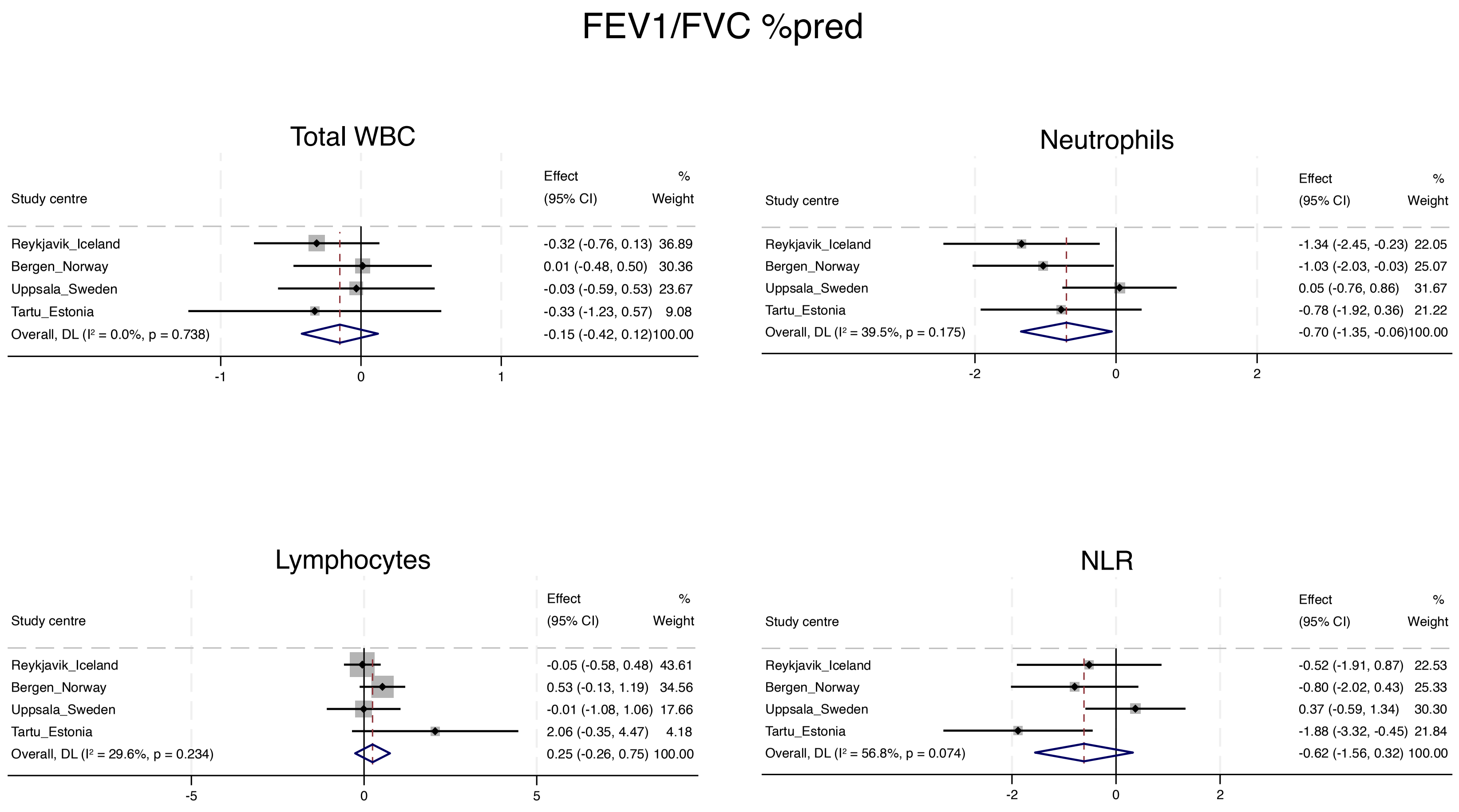
FEV1/FVC %pred
Total WBC
Study centre
Effect (95% CI)
% Weight
Reykjavik_Iceland -0.32 (-0.76, 0.13) 36.89
Bergen_Norway 0.01 (-0.48, 0.50) 30.36
Uppsala_Sweden -0.03 (-0.59, 0.53) 23.67
Tartu_Estonia -0.33 (-1.23, 0.57) 9.08
Overall, DL (I² = 0.0%, p = 0.738) -0.15 (-0.42, 0.12)100.00
-1
0
1
Neutrophils
Study centre
Effect (95% CI)
% Weight
Reykjavik_Iceland -1.34 (-2.45, -0.23) 22.05
Bergen_Norway -1.03 (-2.03, -0.03) 25.07
Uppsala_Sweden 0.05 (-0.76, 0.86) 31.67
Tartu_Estonia -0.78 (-1.92, 0.36) 21.22
Overall, DL (I² = 39.5%, p = 0.175) -0.70 (-1.35, -0.06)100.00
-2
0
2
Lymphocytes
Study centre
Effect (95% CI)
% Weight
Reykjavik_Iceland -0.05 (-0.58, 0.48) 43.61
Bergen_Norway 0.53 (-0.13, 1.19) 34.56
Uppsala_Sweden -0.01 (-1.08, 1.06) 17.66
Tartu_Estonia 2.06 (-0.35, 4.47) 4.18
Overall, DL (I² = 29.6%, p = 0.234) 0.25 (-0.26, 0.75) 100.00
-5
0
5
NLR
Study centre
Effect (95% CI)
% Weight
Reykjavik_Iceland -0.52 (-1.91, 0.87) 22.53
Bergen_Norway -0.80 (-2.02, 0.43) 25.33
Uppsala_Sweden 0.37 (-0.59, 1.34) 30.30
Tartu_Estonia -1.88 (-3.32, -0.45) 21.84
Overall, DL (I² = 56.8%, p = 0.074) -0.62 (-1.56, 0.32) 100.00
-2
0
2

Figure S6.

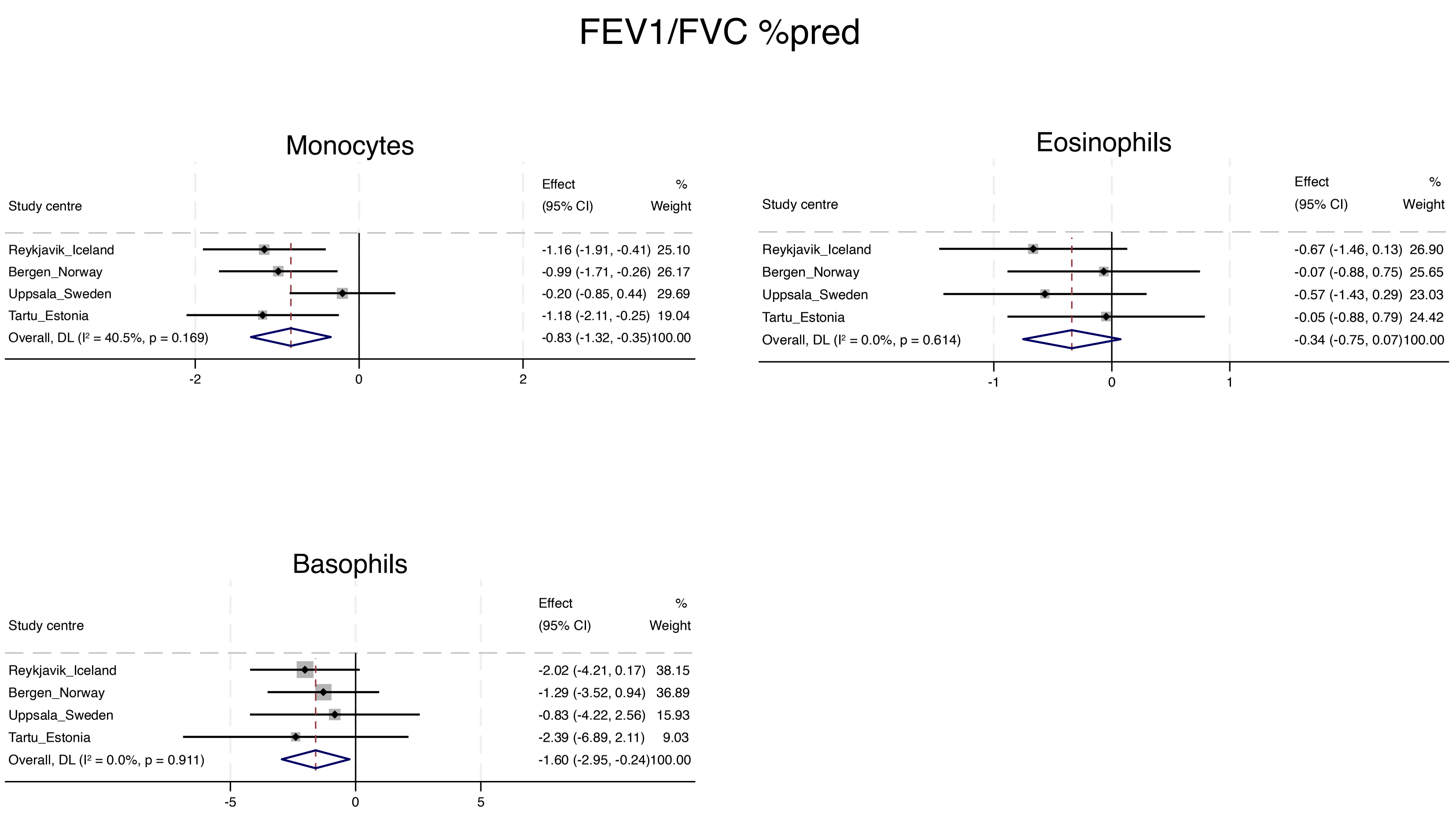

FEV1/FVC %pred
Monocytes
Study centre
Effect (95% CI)
% Weight
Reykjavik_Iceland -1.16 (-1.91, -0.41) 25.10
Bergen_Norway -0.99 (-1.71, -0.26) 26.17
Uppsala_Sweden -0.20 (-0.85, 0.44) 29.69
Tartu_Estonia -1.18 (-2.11, -0.25) 19.04
Overall, DL (I² = 40.5%, p = 0.169) -0.83 (-1.32, -0.35)100.00
-2
0
2
Eosinophils
Study centre
Effect (95% CI)
% Weight
Reykjavik_Iceland -0.67 (-1.46, 0.13) 26.90
Bergen_Norway -0.07 (-0.88, 0.75) 25.65
Uppsala_Sweden -0.57 (-1.43, 0.29) 23.03
Tartu_Estonia -0.05 (-0.88, 0.79) 24.42
Overall, DL (I² = 0.0%, p = 0.614) -0.34 (-0.75, 0.07)100.00
-1
0
1
Basophils
Study centre
Effect (95% CI)
% Weight
Reykjavik_Iceland -2.02 (-4.21, 0.17) 38.15
Bergen_Norway -1.29 (-3.52, 0.94) 36.89
Uppsala_Sweden -0.83 (-4.22, 2.56) 15.93
Tartu_Estonia -2.39 (-6.89, 2.11) 9.03
Overall, DL (I² = 0.0%, p = 0.911) -1.60 (-2.95, -0.24)100.00
-5
0
5

Figure S7.

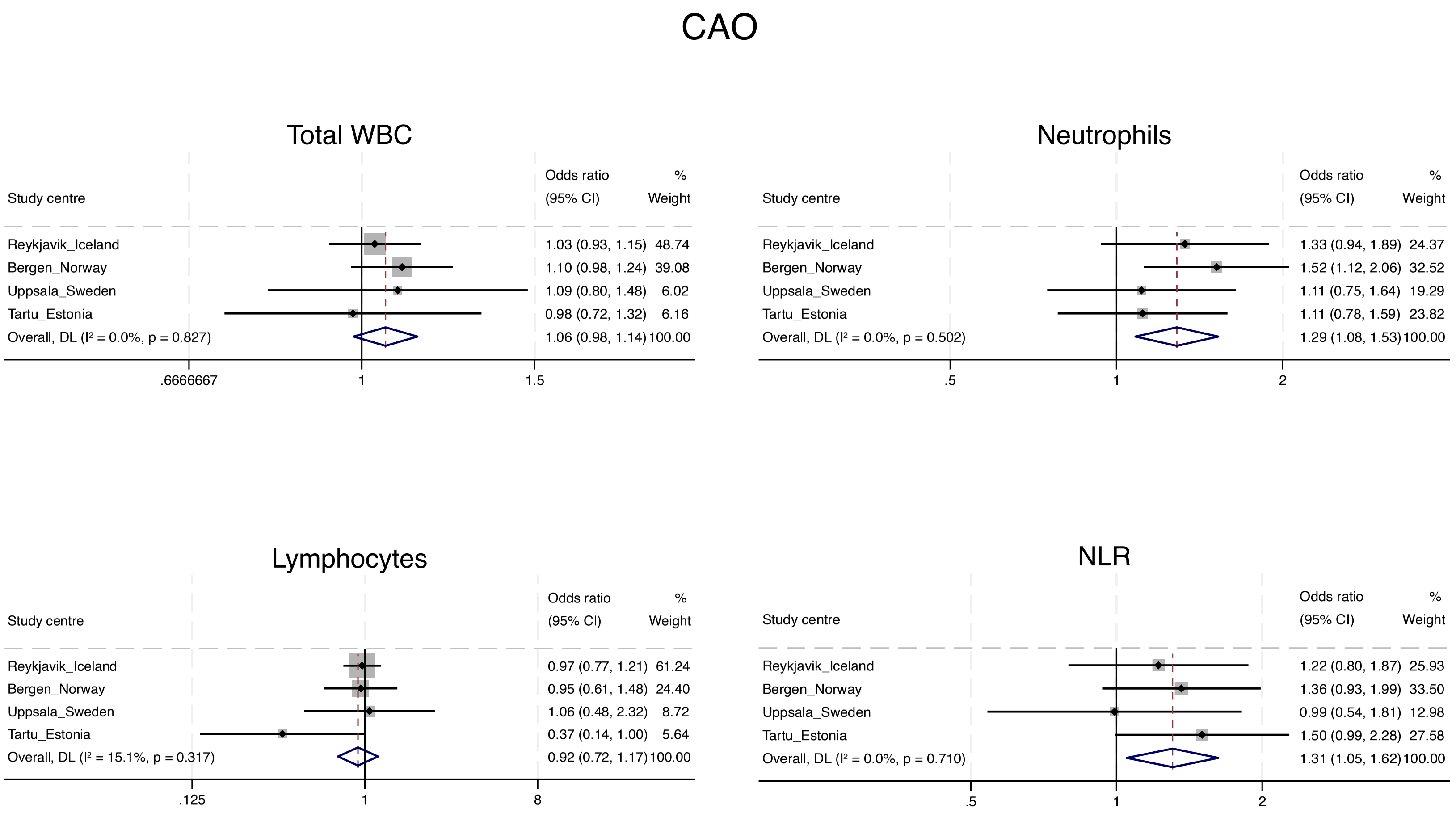
CAO
Total WBC
Study centre
Odds ratio (95% CI)
% Weight
Reykjavik_Iceland 1.03 (0.93, 1.15) 48.74
Bergen_Norway 1.10 (0.98, 1.24) 39.08
Uppsala_Sweden 1.09 (0.80, 1.48) 6.02
Tartu_Estonia 0.98 (0.72, 1.32) 6.16
Overall, DL (I² = 0.0%, p = 0.827) 1.06 (0.98, 1.14) 100.00
.6666667
1
1.5
Neutrophils
Study centre
Odds ratio (95% CI)
% Weight
Reykjavik_Iceland 1.33 (0.94, 1.89) 24.37
Bergen_Norway 1.52 (1.12, 2.06) 32.52
Uppsala_Sweden 1.11 (0.75, 1.64) 19.29
Tartu_Estonia 1.11 (0.78, 1.59) 23.82
Overall, DL (I² = 0.0%, p = 0.502) 1.29 (1.08, 1.53) 100.00
.5
1
2
Lymphocytes
Study centre
Odds ratio (95% CI)
% Weight
Reykjavik_Iceland 0.97 (0.77, 1.21) 61.24
Bergen_Norway 0.95 (0.61, 1.48) 24.40
Uppsala_Sweden 1.06 (0.48, 2.32) 8.72
Tartu_Estonia 0.37 (0.14, 1.00) 5.64
Overall, DL (I² = 15.1%, p = 0.317) 0.92 (0.72, 1.17) 100.00
.125
1
8
NLR
Study centre
Odds ratio (95% CI)
% Weight
Reykjavik_Iceland 1.22 (0.80, 1.87) 25.93
Bergen_Norway 1.36 (0.93, 1.99) 33.50
Uppsala_Sweden 0.99 (0.54, 1.81) 12.98
Tartu_Estonia 1.50 (0.99, 2.28) 27.58
Overall, DL (I² = 0.0%, p = 0.710) 1.31 (1.05, 1.62) 100.00
.5
1
2

Figure S8.

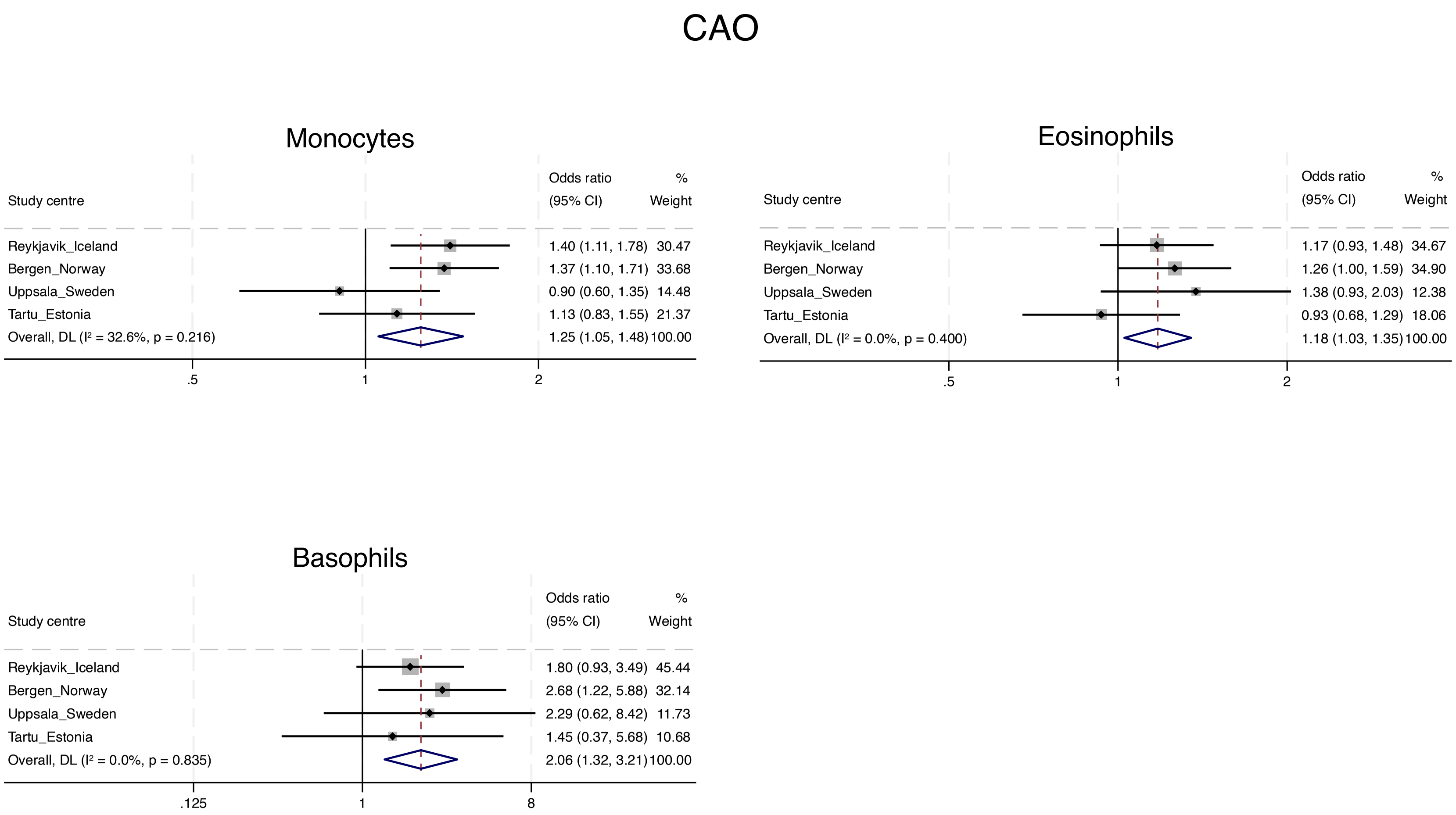
CAO
Monocytes
Study centre
Odds ratio (95% CI)
% Weight
Reykjavik_Iceland 1.40 (1.11, 1.78) 30.47
Bergen_Norway 1.37 (1.10, 1.71) 33.68
Uppsala_Sweden 0.90 (0.60, 1.35) 14.48
Tartu_Estonia 1.13 (0.83, 1.55) 21.37
Overall, DL (I² = 32.6%, p = 0.216) 1.25 (1.05, 1.48) 100.00
.5
1
2
Eosinophils
Study centre
Odds ratio (95% CI)
% Weight
Reykjavik_Iceland 1.17 (0.93, 1.48) 34.67
Bergen_Norway 1.26 (1.00, 1.59) 34.90
Uppsala_Sweden 1.38 (0.93, 2.03) 12.38
Tartu_Estonia 0.93 (0.68, 1.29) 18.06
Overall, DL (I² = 0.0%, p = 0.400) 1.18 (1.03, 1.35) 100.00
.5
1
2
Basophils
Study centre
Odds ratio (95% CI)
% Weight
Reykjavik_Iceland 1.80 (0.93, 3.49) 45.44
Bergen_Norway 2.68 (1.22, 5.88) 32.14
Uppsala_Sweden 2.29 (0.62, 8.42) 11.73
Tartu_Estonia 1.45 (0.37, 5.68) 10.68
Overall, DL (I² = 0.0%, p = 0.835) 2.06 (1.32, 3.21) 100.00
.125
1
8
